# Effects of a school-based cycling intervention on commuting to school behavior and device-measured activity in Spanish adolescents: the PACO cluster-randomized controlled trial

**DOI:** 10.1186/s12966-026-01893-1

**Published:** 2026-02-24

**Authors:** Pablo Campos-Garzón, F. Javier Huertas-Delgado, Cristina Cadenas-Sanchez, Javier Molina-García, Yaira Barranco-Ruiz, Palma Chillón

**Affiliations:** 1 Department of Global Public Health, Karolinska Institutet, Stockholm, Sweden; 2 Department of Physical Education and Sports, Faculty of Sports Science, Sport and Health University Research Institute (iMUDS), University of Granada, Ctra. Alfacar, s/n, Granada, 18011 Spain; 3 Teacher Training Centre La Inmaculada, Universidad de Granada, Granada, España; 4 CIBER de Fisiopatología de la Obesidad y Nutrición (CIBEROBN), Instituto de Salud Carlos III, Granada, Spain; 5 AFIPS Research Group, Department of Teaching of Physical Education, Arts and Music, University of Valencia, Valencia, Spain; 6 Epidemiology and Environmental Health Joint Research Unit, FISABIO-UJI-UV, Valencia, España

**Keywords:** Active transport, Basic psychological needs, Barriers, Motivation, Public health

## Abstract

**Background:**

Active commuting to/from school (ACS) is associated with multiple health and societal benefits, yet school-based interventions have shown limited success in changing adolescents’ commuting behavior, and their effects on psychosocial factors remain unclear. This study primarily examined the effects of a school-based cycling intervention on the usual mode and frequency of ACS, and ACS-related psychosocial outcomes in Spanish adolescents. Secondary outcomes included device-measured sedentary time and physical activity (PA).

**Methods:**

A cluster-randomized controlled trial was conducted in eight Spanish secondary schools. A total of 256 adolescents (45.7% girls; mean age 14.4 years) were allocated to an intervention (*n* = 127) or control group. The intervention consisted of four weekly sessions delivered during Physical Education classes over one month, combining cycling theory, skills training in closed circuits, and supervised on-road cycling in urban environments. Outcomes were assessed at baseline and post-intervention and included usual mode and weekly frequency of ACS, perceived barriers to ACS, basic psychological need satisfaction in ACS, motivation for ACS, and device-measured sedentary time and PA across daily segments.

**Results:**

No significant between-group effects were observed for usual mode or frequency of ACS, nor for device-measured sedentary time or PA. Most psychosocial outcomes did not differ between groups. However, perceived environmental/safety barriers increased in the intervention group compared with controls (Δ = 0.22, *p* = 0.041). Moderation analyses showed that girls in the intervention group reported greater increases in amotivation for ACS than girls in the control group (Δ = 0.54, *p* = 0.018). Per-protocol analyses revealed higher external motivation (Δ = 0.40, *p* = 0.029) and amotivation (Δ = 0.38, *p* = 0.037) in the intervention group, with stronger effects among girls and adolescents from higher socioeconomic backgrounds.

**Conclusions:**

The school-based cycling program did not change the commuting behavior or device-measured activity. Instead, participation was associated with increased awareness of environmental/safety barriers and higher amotivation, particularly among girls. Per-protocol analyses also revealed increases in external motivation and perceived barriers, particularly among girls and high-SES adolescents. These findings suggest that short-duration, skills-focused cycling interventions may heighten perceived constraints without being sufficient to support behavior change. Future programs may require longer duration, autonomy-supportive delivery, and complementary built environmental and family-level strategies to effectively promote ACS among adolescents.

**Trial registration:**

ClinicalTrials.gov (Identifier: NCT03937336).

**Supplementary Information:**

The online version contains supplementary material available at 10.1186/s12966-026-01893-1.

## Background

Regular physical activity (PA) is associated with better physical, mental, and social health in adolescents [1, 2]. Nevertheless, adolescent PA remains insufficient globally with minimal progress over the last decade [3], alongside pronounced gender and socioeconomic inequities [4, 5]. In this context, active commuting to and from school (ACS), mainly walking and cycling [6], represents a scalable source of daily PA [7]. Beyond PA, it may also improve other health indicators (e.g., body composition, fitness) [8], and yields societal co-benefits such as reduced emissions and congestion [9].

Despite well-documented health benefits, ACS prevalence has declined in many countries [10–12]. In Spain, however, ACS has remained relatively stable over 2010–2017 [13] yet cycling to school remains uncommon (< 1%; [14]). This is striking given that ACS, and cycling in particular, is linked to higher PA and fitness and better health profiles in youth [8, 15]. Numerous school-based programs have been trialed to reverse these patterns, but effects are generally trivial-to-small [16–18]. Most interventions have prioritized shifts in the mode of commuting or the number of active trips as primary endpoints [19–21]. Nevertheless, an intervention can be successful even without an immediate shift in mode of commuting if it reduces perceived barriers, enhances basic psychological needs satisfaction (BPNS), or strengthens autonomous motivation [22–24]. Such changes indicate greater capability and readiness for ACS and may lay the groundwork for a durable behavior change. Regarding PA, many studies implicitly assume that a shift in commuting mode leads to higher daily activity. However, evidence on whether mode of commuting shifts translate into higher daily PA is inconsistent, with studies reporting increases [25–27], no changes [21, 28], or even decreases [29]. These inconsistencies emphasize the need for school-based cycling interventions to clarify their potential effects among adolescents.

Therefore, this study aimed to analyze the effects of a school-based cycling intervention on the usual mode and frequency of ACS, as well as ACS-related psychosocial outcomes (perceived barriers to ACS [i.e., environmental and safety, planning and psychosocial], BPNS related to ACS [i.e., autonomy, competence, and relatedness], and motivation for ACS [i.e., intrinsic, integrated, identified, introjected, external, and amotivation]). Secondary outcomes included device-measured sedentary time and PA levels (i.e., light PA [LPA] and moderate-to-vigorous PA [MVPA] during the total day, weekdays, out-of-school periods, and weekend days) in Spanish adolescents.

## Methods

### Study design

The PACO study is a cluster-randomized controlled trial which was conducted in four Spanish cities (i.e., Almería, Granada, Jaén, and Valencia), with two arms (i.e., intervention and control groups). Secondary schools served as the unit of randomization. The primary aim of the PACO study was to promote cycling to and from school and PA in adolescents, through a school-based cycling intervention. The intervention was based on the Bikeability framework, an evidence-based cycling education approach widely implemented in school settings [30], and was delivered over four weekly sessions intentionally designed to ensure feasibility within the Physical Education curriculum. Outcomes were assessed at baseline and immediately after the four-week intervention period. The full PACO study protocol and a detailed description of the intervention have been published elsewhere [31]. The trial was registered at ClinicalTrials.gov (Identifier: NCT03937336) and conducted in accordance with the CONSORT guidelines for cluster-randomized trials. The study protocol was approved by the Review Committee for Research Involving Human Subjects at the University of Granada (Reference: 162/CEIH/2016).

### Participants

**Fig. 1:**
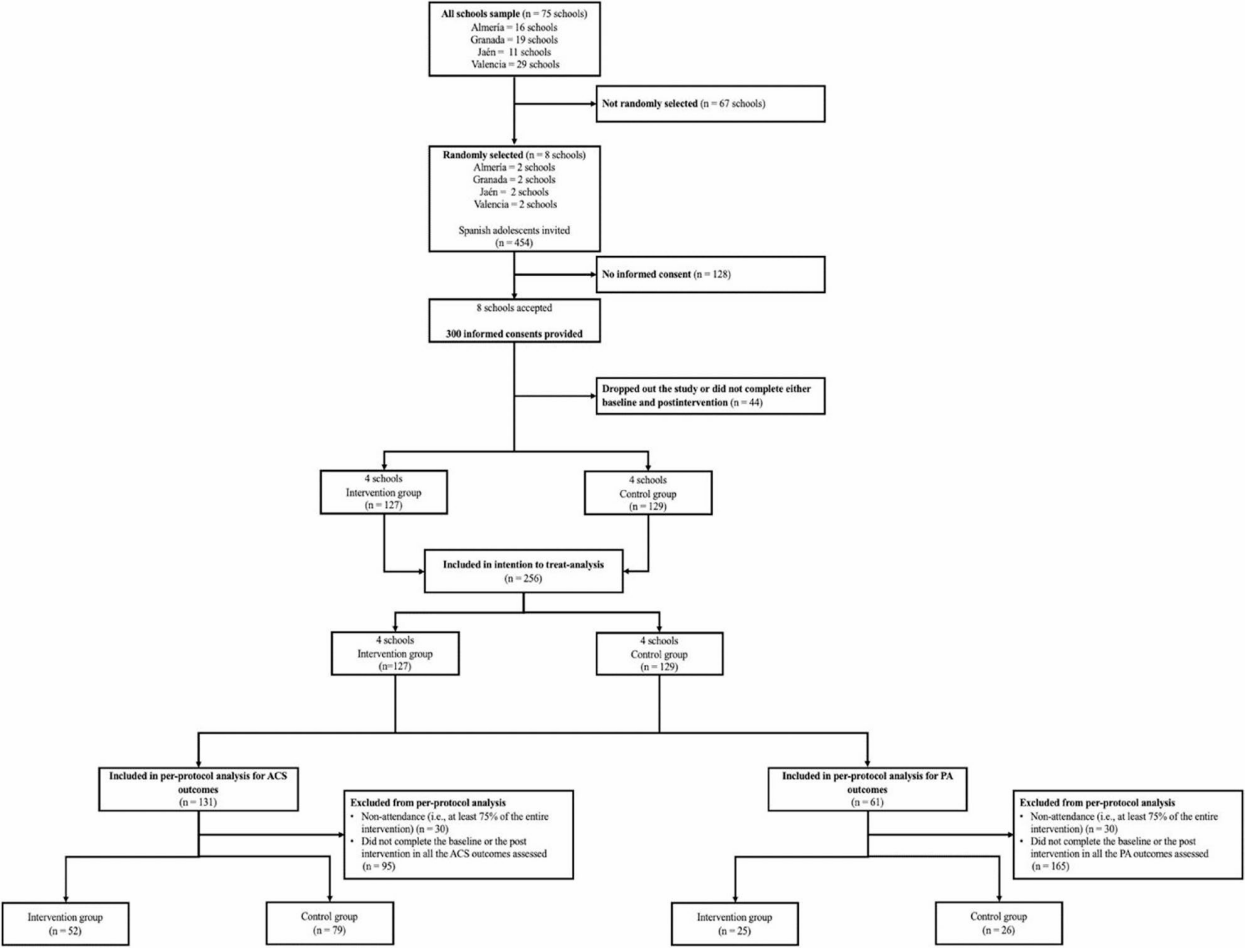
Flow chart of the participants

Eligible participants were adolescents enrolled in the 3rd grade of secondary education. Inclusion criteria for school participation were: (1) having at least two classes of 3rd grade with ≥ 15 adolescents per class; (2) obtaining a minimum of 15 signed parental consent forms per class; (3) no prior involvement of students in other interventions promoting PA or exercise during the PACO study intervention period; and (4) no provision of school transport to students. Adolescents were excluded if they were unable to attend Physical Education lessons (e.g., due to physical or mental health conditions) at the time of data collection. A total of 454 students were invited to participate, of whom 300 returned signed parental consent forms. Of these, 256 provided data at either baseline or follow-up and were included in the intention-to-treat analyses (Fig. 1).

### Recruitment and randomization

A list of all public high schools from each participating city was obtained from the corresponding regional educational authorities. Schools were randomly selected in each city, and this random selection was repeated to create an ordered list of all the public high schools participating. In each city, one school was allocated to the intervention group and one to the control group. If a selected school declined participation, the same protocol was applied to the next school in the ordered list. Recruitment of students was conducted by research staff during visits to the selected classes, where the study was explained and consent forms were distributed. Only adolescents with signed parental consent were included in the study.

### Data collection

Data were collected from January 2018 to June 2021. Due to a shortage of devices (i.e., accelerometers), only two schools could be assessed simultaneously. Baseline and follow-up assessments included a school visit for distribution of belts equipped with accelerometers to be worn for seven consecutive days. After one week, research staff returned to administer questionnaires and collected the devices. It is important to note that data collection was affected by the COVID-19 pandemic due to public health restrictions in Spain. As a result, the device-measured in the follow-up was lost in the participants from Valencia, and the questionnaire which was originally planned in paper format was administered online.

### Measures

#### Primary outcomes

##### Mode and frequency of ACS

Mode of commuting to and from school was assessed with a reliable and validated instrument, “the PACO questionnaire” [32, 33]. Four items assessed the usual and daily (previous week) modes of commuting to and from school. Response options included walking, cycling, car, motorcycle, school bus, public bus, metro/train/tram, or other. For the usual mode of commuting, walking and cycling were classified as active, while all other modes were classified as passive. The total number of weekly walking and cycling trips was derived from the daily responses.

##### ACS-related psychosocial outcomes

***Perceived barriers to ACS***

Perceived barriers were measured with the Barreras en el Transporte Activo al Centro Educativo questionnaire [34]. Two categories of barriers were assessed: environmental/safety and planning/psychosocial. Example items include *“There is too much traffic on the way to school”*, *“I would have to walk or cycle through unsafe areas because of crime or vandalism”*. Each category was scored as a continuous variable ranging from 1 (lowest possible perception of barriers) to 4 (highest possible perception).

***BPNS related to ACS***

Autonomy, competence, and relatedness related to ACS were assessed using the Basic Psychological Need Satisfaction in ACS scale [34]. Example items include *“I can choose how I travel to school”*, *“I feel skilled at travelling to school walking or cycling”*, *“I get al.ong very well with the people who accompany me on the way to school”*. Each need was scored as a continuous variable ranging from 0 (lowest possible perceived satisfaction) to 4 (highest possible perceived satisfaction).

***Motivation for ACS***

Motivation for ACS was measured with the Behavioural Regulation in ACS questionnaire [35], which assesses six types of motivation: intrinsic, integrated, identified, introjected, external, and amotivation. Example items include *“I walk or cycle to school because I enjoy it”*, *“I value the benefits of going to school walking or cycling”*, *“I think that going to school walking or cycling is a waste of time”*. Each dimension was scored as a continuous variable ranging from 1 (lowest possible motivation) to 5 (highest possible motivation).

***Sedentary time and PA levels***

Sedentary time and PA were measured using triaxial accelerometers (ActiGraph GT3x+, Pensacola, FL, USA). Devices were initialized at 90 Hz and processed using the open-source R package GGIR v.3.0–0 [36]. Data processing included auto-calibration [36], non-wear detection and imputation [37], and conversion of raw signals into activity counts over 15-second epochs [38]. Cut-points from Evenson et al. [39] were applied: 0–26 counts/15s for sedentary time, 26–574 counts/15s for LPA, and ≥ 574 counts/15s for MVPA.

Moreover, sedentary time and PA levels were estimated across different time segments: total day (daily average), weekdays (school days), in-school hours (according to each center’s schedule), out-of-school hours (all weekday time outside school), and weekends. These filters were applied during data processing using the open-source R package GGIR (v3.0-0) [36].

### Covariates

Participants completed a self-administered questionnaire in which they reported basic demographic information, such as age and gender, and if they owned a bike in good condition. Socioeconomic status (SES) was assessed using a modified version of the Family Affluence Scale II [40]. Home-to-school distance was calculated using the geographic information system QGIS 3.28.8 as the shortest network distance from each participant’s self-reported home postal address to the school.

### Intervention program

The school-based cycling intervention had a duration of four weeks, one session per week, and it followed the Bikeability methodology [30]. The intervention was delivered by members of the research staff with experience in Physical Education and cycling training. The four intervention sessions consisted in: (1) Theoretical Session (60 min - first week): In this session, participants learnt about the importance and the benefits of cycling as a mode of commuting, as well as they received information about road safety rules, equipment, and hand signals in urban contexts; (2) Closed Circuit Session (120 min - second week): Participants had a practical session on the school grounds to learn the fundamental cycling skills necessary to ride safely. They practiced fitting their helmet, checking their bike, and important skills such as starting off, pedaling, braking, changing gears, and signaling; (3) Urban Circuit Session (120 min - third week): In this practical session on-road traffic, participants applied their previously learned skills in new and uncertain situations. They experienced urban traffic scenarios such as starting from the side of the road, overtaking slow-moving vehicles, lane changing, and crossing a roundabout; and (4) Bicycle’s Party (120 min - fourth week): This final session took place on the school grounds, and it was designed to reinforce previous learning. Participants became teachers to younger peers and engaged in activities such as bike fitting, helmet fitting, basic bicycle checks, cycling rules and signaling, a basic cycling skills circuit, and exercises to develop the main skills required for road traffic. This last session was replaced by a second urban circuit session during Covid times, since students from different classes could not be mixed to avoid contacts. Moreover, schools allocated to the control group continued with their usual curriculum and did not receive any cycling-related, active commuting, or PA intervention during the study period. After completion of the post-intervention assessments, control schools were offered the intervention materials and the opportunity to implement the cycling sessions at their own schools.

### Statistical analysis

Descriptive characteristics of the sample are presented as mean ± standard deviation (SD) or as frequencies and percentages, as appropriate.

The main effects of the PACO study were tested with the intention-to-treat approach. Although the original study protocol, published in 2021, described the use of analysis of covariance (ANCOVA), intervention effects have been analyzed using linear mixed-effects models, which provide a more appropriate framework for cluster-randomized longitudinal data by accounting for the hierarchical structure of the data (schools and participants) and repeated measurements over time [41]. A series of linear mixed-effects models were fitted with fixed effects for group (intervention vs. control), time (baseline vs. follow-up), and their interaction. Random intercepts were specified for participants nested within schools to account for the clustered and longitudinal structure of the data. Exploratory moderation analyses by gender (boys, girls) and SES (high and medium categories, with low-SES cases incorporated into the medium category due to low frequency) were conducted, motivated by prior evidence suggesting differential responses to ACS interventions [42], by including three-way interaction terms (group × time × moderator). No data imputation was conducted [43], as baseline measurements were treated as components of the outcome variables, and all participants with at least one available assessment were included in the analysis [41]. Missing values in post-intervention outcomes (both primary and secondary) were due to study withdrawal prior to completion and were assumed to be missing at random, given the absence of significant differences between participants who dropped out and those who completed the study.

Data were presented as means and differences in the mean changes with 95% confidence interval as an indicator of variance. The adequacy of the linear mixed-effects models was assessed by inspecting predicted values and the distribution of model residuals using graphical procedures (e.g., Q–Q plots). The following covariates were included in the models: gender, owning a bike in good conditions, SES, home-school distance, and wear time for accelerometry analysis. The estimates were presented both as raw values and as standardized mean differences (Cohen’s d). Standardized scores were calculated relative to the baseline mean and standard deviation. Effect sizes were interpreted as small (0.2 SD), medium (0.5 SD), and large (0.8 SD) [44].

Regarding sensitivity analysis, per-protocol analyses were presented as supplementary material and followed the same procedure as the explained above for the intention-to-treat (ITT) analyses. Briefly, the inclusion criteria for the per-protocol approach were: (1) to have valid data in both pre- and post-intervention assessments and (2) to attend at least 75% of the intervention sessions (intervention group only), corresponding to a minimum of three out of four sessions.

All analyses were performed using R software version 4.3.1 and RStudio version 2023.09.0 + 463. For inferential statistics, the level of statistical significance was set at p *<* 0.05. Moreover, multiple comparisons for both primary and secondary outcomes were adjusted using the false discovery rate approach described by Benjamini & Hochberg [45].

## Results

### Descriptive data

Descriptive characteristics of the participants at baseline are presented in Table 1. The total sample included 256 adolescents (53.1% girls) with a mean age of 14.4 ± 0.6 years. Approximately 55% of participants actively commuted to school, and 57% reported active commuting from school. The percentage of students owning a bike in good condition was higher in the intervention group compared to the control group (55.9% vs. 42.6%). In both groups, participants lived farther from 3.0 km from school (3.7 ± 1.1 km and 3.3 ± 4.3 km, respectively). Overall, participants reported medium scores for perceived barriers to ACS (range: 0–4), BPNS related to ACS (range: 0–5), and motivation for ACS (range: 0–4), except for introjected regulation, external regulation, and amotivation, which showed low scores. Moreover, Table 2 presents accelerometer-derived estimates of wear time, sedentary time, LPA, and MVPA across different time contexts. Overall, participants spent most of their time engaged in sedentary behaviors across all time contexts assessed. Regarding PA levels, both LPA and MVPA were higher during the total day and on weekdays, but lower during out of school periods and on weekends.

**Table 1:** Descriptive characteristics of the participants in the PACO study at baseline

	All (*n* = 256)	Intervention group (*n* = 127)	Control group (*n* = 129)
Age (years)	14.4 ± 0.6	14.3 ± 0.6	14.4 ± 0.7
Girls (%)	136 (53.1)	58 (45.7)	78 (60.5)
Usual mode of commuting			
Active commuting to school (%)	141 (55.1)	71 (55.9)	70 (54.3)
Active commuting from school (%)	146 (57.0)	73 (57.5)	73 (56.6)
Weekly frequency of ACS			
Walking (nº trips)	5.6 ± 4.3	5.9 ± 3.8	5.3 ± 4.6
Cycling (nº trips)	0.2 ± 1.3	0.3 ± 1.6	0.2 ± 1.0
*Bike in good condition*			
Yes	162 (63.3)	71 (55.9)	55 (42.6)
No	93 (36.7)	55 (54.1)	38 (29.5)
SES			
Medium (%)	111 (43.4)	50 (39.4)	61 (47.3)
High (%)	145 (56.6)	77 (60.6)	38 (52.7)
Home-school distance (km)	3.5 ± 8.2	3.7 ± 1.1	3.3 ± 4.3
Perceived barriers to ACS			
Environmental/safety barriers (score)	1.8 ± 0.7	1.8 ± 0.6	1.8 ± 0.7
Planning/psychosocial barriers (score)	1.9 ± 0.6	1.8 ± 0.6	1.9 ± 0.7
Basic psychological needs related to ACS			
Autonomy (score)	3.4 ± 1.1	4.1 ± 0.9	3.6 ± 1.1
Competence (score)	4.1 ± 1.3	4.3 ± 1.1	3.9 ± 1.4
Relatedness (score)	4.4 ± 0.9	4.4 ± 0.9	4.4 ± 0.9
Motivation for ACS			
Intrinsic (score)	2.4 ± 1.4	2.5 ± 1.3	2.3 ± 1.4
Integrated (score)	2.0 ± 1.4	2.1 ± 1.3	1.8 ± 1.4
Identified (score)	2.2 ± 1.3	2.2 ± 1.3	2.1 ± 1.4
Introjected (score)	0.5 ± 0.7	0.4 ± 0.6	0.5 ± 0.7
External (score)	0.6 ± 0.9	0.5 ± 0.8	0.7 ± 0.9
Amotivation (score)	0.7 ± 0.9	0.6 ± 0.8	0.8 ± 0.9

Results are presented as mean ± standard deviation for continuous variables and as n (%) for categorical outcomes

Basic psychological needs related to ACS ranges from 0 (lower basic psychological needs related to ACS) to 5 (higher basic psychological needs related to ACS); perceived barriers to ACS ranges from1 (lower perception of barriers to ACS) to 4 (higher perception of barriers to ACS; motivation for ACS ranges from 0 (lower motivation for ACS) to 4 (higher motivation for ACS), it is important to note that amotivation’s interpretation is inversely

*n* Sample size, *%* Percentage, *SES* Socioeconomic status, *km* Kilometer; *nº* Number, *ACS* Active commuting to and from school

**Table 2:** Device-measured sedentary time and PA levels across total day, weekdays, out-of-school, and weekends at baseline

	All (*n* = 256)	Intervention group (*n* = 127)	Control group (*n* = 129)
Total day			
Wear time (min)	790.5 ± 113.4	777.0 ± 114.7	884.9 ± 110.6
Sedentary time (min)	597.1 ± 108.3	582.8 ± 109.9	612.5 ± 104.9
LPA (min)	155.9 ± 47.8	156.6 ± 58.4	155.1 ± 47.3
MVPA (min)	37.5 ± 22.5	37.6 ± 25.5	37.3 ± 22.5
Weekdays			
Wear time (min)	809.9 ± 125.2	793.4 ± 123.4	827.7 ± 124.1
Sedentary time (min)	611.5 ± 118.1	591.5 ± 117.5	633.1 ± 115.3
LPA (min)	158.9 ± 51.1	161.5 ± 53.1	156.2 ± 49.0
MVPA (min)	39.5 ± 23.4	40.5 ± 23.9	38.4 ± 22.8
Out of school			
Wear time (min)	717.0 ± 224.2	694.6 ± 204.0	741.1 ± 242.7
Sedentary time (min)	352.8 ± 120.6	334.8 ± 110.7	372.7 ± 128.2
LPA (min)	144.5 ± 47.3	147.4 ± 45.4	141.3 ± 49.3
MVPA (min)	32.7 ± 20.2	31.9 ± 20.7	33.5 ± 19.7
Weekends			
Wear time (min)	721.2 ± 140.4	716.2 ± 141.0	725.5 ± 140.5
Sedentary time (min)	543.7 ± 135.8	543.7 ± 125.1	543.6 ± 145.2
LPA (min)	147.0 ± 55.2	145.6 ± 54.5	148.3 ± 56.0
MVPA (min)	30.5 ± 30.5	26.9 ± 26.4	33.5 ± 33.6

Results are presented as mean ± standard deviation

*n* Sample size, *min* Minutes, *LPA* Light physical activity, *MVPA* Moderate-to-vigorous physical activity

### ITT analysis: primary outcomes

**Fig. 2:**
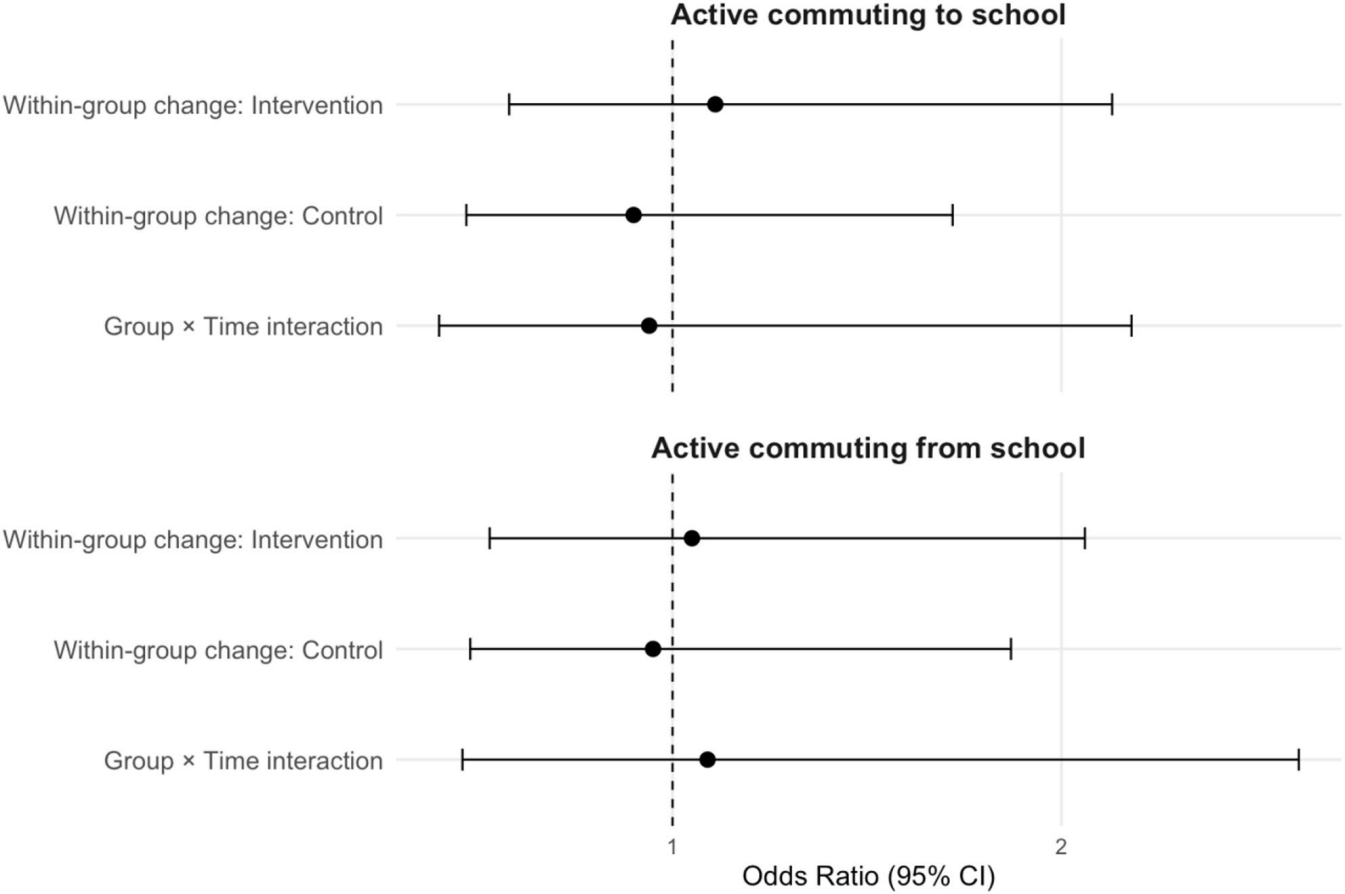
Within- and between-group changes in the usual mode of commuting to and from school from baseline to follow-up. Odds ratios (OR) and 95% confidence intervals for between-group, within-group, and interaction effects. Reference: passive commuting. Adjusted for: gender, bike in good condition, SES, and home-school distance

Figure 2 displays the changes in the usual mode of commuting (active vs. passive) between baseline and follow-up within and between groups (intervention vs. control). No significant within-group changes from baseline to follow-up nor the group × time interaction terms were observed (all, p> 0.05).

Changes in ACS-related outcomes over time and between groups are presented in Table 3. No significant between-group effects were observed for the frequency of ACS or for ACS-related psychosocial outcomes, except for perceived environmental/safety barriers, which increased in the intervention group compared with the control group after the intervention (mean difference = 0.22; *p* = 0.041; d = 0.26).

**Table 3:** Effects of the intervention on weekly frequency of ACS, basic psychological needs related to ACS, perceived barriers to ACS, and motivational outcomes for ACS from baseline to follow-up

Outcome	Intervention group Mean (95% CI) (*n* = 127) (Follow-up minus baseline)	Control group Mean (95% CI) (*n* = 129) (Follow-up minus baseline)	Difference in change from baseline to follow-up Mean (95% CI) (Intervention minus control)	Group X time *p*-value
**Weekly frequency of ACS**				
*Walking trips*				
Number of trips	0.55 (-0.18 to 1.29)	0.29 (-0.40 to 0.98)	0.26 (-0.74 to 1.27)	0.264
Cohen’s d	0.05 (-0.20 to 0.30)	0.03 (-0.22 to 0.27)	0.06 (-0.18 to 0.31)	
*Cycling trips*				
Number of trips	-0.02 (-0.27 to 0.22)	0.07 (-0.15 to 0.30)	-0.10 (-0.44 to 0.23)	0.552
Cohen’s d	-0.01 (-0.26 to 0.23)	0.03 (-0.21 to 0.28)	-0.07 (-0.32 to 0.18)	
**Perceived barriers to ACS**				
*Environmental/safety barriers*				
Score	**0.20 (0.04 to 0.36)**	-0.02 (-0.17 to 0.12)	**0.22 (0.01 to 0.44)**	0.041
Cohen’s d	**0.25 (0.01 to 0.50)**	-0.03 (-0.27 to 0.22)	**0.26 (0.01 to 0.51)**	
*Planning/psychosocial barriers*				
Score	0.14 (-0.01 to 0.29)	0.08 (-0.08 to 0.22)	0.06 (-0.14 to 0.27)	0.530
Cohen’s d	0.16 (-0.09 to 0.40)	0.09 (-0.16 to 0.33)	0.07 (-0.18 to 0.31)	
**Basic psychological needs related to ACS**				
*Autonomy*				
Score	0.05 (-0.14 to 0.24)	0.12 (0.09 to -0.06)	-0.07 (-0.32 to 0.19)	0.602
Cohen’s d	0.04 (-0.20 to 0.29)	0.09 (-0.16 to 0.33)	-0.01 (-0.25 to 0.24)	
*Competence*				
Score	-0.10 (-0.32 to 0.13)	0.08 (-0.13 to 0.30)	-0.18 (-0.49 to 0.13)	0.251
Cohen’s d	-0.07 (-0.31 to 0.18)	0.05 (-0.19 to 0.30)	-0.18 (-0.42 to 0.17)	
*Relatedness*				
Score	-0.16 (-0.35 to 0.03)	-0.11 (-0.29 to 0.07)	-0.05 (-0.32 to 0.21)	0.694
Cohen’s d	-0.12 (-0.37 to 0.13)	-0.08 (-0.32 to 0.17)	-0.05 (-0.29 to 0.20)	
**Motivation for ACS**				
*Intrinsic motivation*				
Score	0.11(-0.15 to 0.38)	0.18 (-0.06 to 0.43)	-0.07 (-0.43 to 0.29)	0.698
Cohen’s d	0.07 (-0.18 to 0.31)	0.10 (-0.15 to 0.34)	-0.05 (-0.29 to 0.20)	
*Integrated motivation*				
Score	0.07 (-0.20 to 0.34)	0.24 (-0.02 to 0.49)	-0.17 (-0.54 to 0.20)	0.367
Cohen’s d	0.02 (-0.22 to 0.27)	0.07 (-0.17 to 0.32)	-0.11 (-0.36 to 0.13)	
*Identified motivation*				
Score	0.01 (-0.27 to 0.26)	0.12 (-0.12 to 0.37)	-0.13 (-0.49 to 0.23)	0.480
Cohen’s d	0.00 (-0.25 to 0.25)	0.08 (-0.17 to 0.32)	-0.09 (-0.34 to 0.15)	
*Introjected motivation*				
Score	**0.40 (0.17 to 0.62)**	**0.28 (0.07 to 0.49)**	0.12 (-0.19 to 0.42)	0.459
Cohen’s d	**0.35 (0.11 to 0.60)**	0.24 (-0.01 to 0.48)	0.11 (-0.14 to 0.35)	
*External motivation*				
Score	**0.27 (0.05 to 0.50)**	0.11 (-0.10 to 0.32)	0.16 (-0.15 to 0.47)	0.312
Cohen’s d	0.24 (-0.01 to 0.49)	0.08 (-0.16 to 0.33)	0.13 (-0.13 to 0.37)	
*Amotivation*				
Score	**0.38 (0.14 to 0.61)**	0.05 (-0.18 to 0.28)	0.33 (-0.01 to 0.66)	0.050
Cohen’s d	**0.27 (0.02 to 0.52)**	0.03 (-0.21 to 0.28)	0.24 (-0.01 to 0.48)	

*ACS* Active commuting to and from school, *CI* Confidence interval, *n* Sample size

All data presented were adjusted for gender, bike in good condition, SES and home-school distance. Bold values indicate significance (*p* < 0.05)

### ITT analysis: Secondary outcomes

Changes in device-measured sedentary time and PA are presented in Table 4. No significant group-by-time interaction effects were found for sedentary time, LPA, or MVPA across any of the time contexts assessed (all *p* > 0.05).

**Table 4:** Effects of the intervention on daily device-measured sedentary time and physical activity, during weekdays, out of school, and during weekends from baseline to follow-up

Outcome	Intervention group Mean (95% CI) (*n* = 127) (Follow-up minus baseline)	Control group Mean (95% CI) (*n* = 129) (Follow-up minus baseline)	Difference in change from baseline to follow-up Mean (95% CI) (Intervention minus control)	Group X time *p*-value
Total day				
Sedentary time (min)	12.00 (-2.76 to 26.80)	4.09 (-9.66 to 17.83)	7.90 (-12.13 to 28.02)	0.437
Cohen’s d	0.07 (-0.18 to 0.33)	0.04 (-0.22 to 0.29)	0.10 (0.15 to 0.35)	
LPA (min)	-9.11 (-20.6 to 2.39)	-4.40 (-15.14 to 6.26)	-4.71 (-20.38 to 10.84)	0.550
Cohen’s d	-0.10 (-0.35 to 0.16)	0.04 (-0.30 to 0.21)	-0.07 (-0.32 to 0.17)	
MVPA (min)	-0.49 (-6.58 to 5.61)	-0.21 (-5.84 to 5.42)	-0.28 (-8.52 to 7.97)	0.948
Cohen’s d	-0.01 (-0.25 to 0.24)	-0.01 (-0.25 to 0.24)	-0.01 (-0.25 to 0.24)	
Weekdays				
Sedentary time (min)	9.92 (-7.24 to 26.13)	0.28 (-14.58 to 15.17)	9.64 (-12.15 to 31.43)	0.383
Cohen’s d	0.06 (-0.19 to 0.31)	0.00 (-0.25 to 0.25)	0.11 (-0.14 to 0.35)	
LPA (min)	-9.71 (-21.89 to 2.42)	-1.02 (-12.32 to 10.21)	-8.69 (-25.11 to 7.76)	0.297
Cohen’s d	-0.11 (-0.36 to 0.14)	-0.01 (-0.26 to 0.23)	-0.13 (-0.38 to 0.12)	
MVPA (min)	-0.53 (-7.12 to 6.06)	0.97 (-5.07 to 7.00)	-1.50 (-10.46 to 7.37)	0.739
Cohen’s d	-0.01 (-0.25 to 0.24)	0.01 (-0.23 to 0.26)	-0.04 (-0.29 to 0.20)	
Out of school				
Sedentary time (min)	1.55 (-32.20 to 35.31)	27.83 (-1.65 to 57.32)	-26.37 (-69.44 to 16.84)	0.231
Cohen’s d	0.00 (-0.24 to 0.25)	0.12 (-0.13 to 0.37)	-0.14 (-0.39 to 0.10)	
LPA (min)	**62.91 (43.60 to 82.22)**	**48.17 (31.15 to 65.02)**	14.8 (-9.71 to 39.47)	0.235
Cohen’s d	**0.61 (0.35 to 0.86)**	**0.48 (0.23 to 0.73)**	0.14 (-0.10 to 0.39)	
MVPA (min)	7.97 (-4.56 to 20.50)	-1.49 (-11.97 to 8.98)	9.46 (-6.59 to 25.50)	0.246
Cohen’s d	0.10 (-0.14 to 0.35)	-0.02 (-0.27 to 0.22)	0.14 (-0.10 to 0.39)	
Weekend				
Sedentary time (min)	1.83 (-24.35 to 28.03)	-0.71 (-24.45 to 23.08)	2.55–32.87 to 37.94)	0.887
Cohen’s d	0.01 (-0.23 to 0.26)	-0.01 (-0.25 to 0.24)	0.02 (-0.23 to 0.26)	
LPA (min)	-3.83 (-23.81 to 16.17)	2.65 (-15.42 to 20.77)	-6.47 (-33.40 to 20.52)	0.635
Cohen’s d	-0.03 (-0.28 to 0.21)	0.02 (-0.22 to 0.27)	-0.06 (-0.31 to 0.19)	
MVPA (min)	2.40 (-9.26 to 14.05)	-1.69 (12.13 to 8.74)	4.09 (-11.68 to 19.83)	0.607
Cohen’s d	0.04 (-0.21 to 0.28)	-0.03 (-0.27 to 0.22)	0.06 (-0.18 to 0.31)	

*CI* Confidence interval, *LPA* Light physical activity, *MVPA* Moderate-to-vigorous physical activity, *min* Minutes, *n* Sample size

All data presented were adjusted for gender, bike in good condition, SES, home-school distance, and wear time in each examined context (i.e., total wear time, weekdays wear time, out of school wear time, and weekends wear time). Bold values indicate significance (*p* < 0.05)

### ITT: moderation analysis

Regarding the moderation analysis, no significant three-way interaction was observed between allocation group, time point, and gender (Table S3, Figures S1, S2, and S5), or between allocation group, time point, and SES (Figures S3, S4, and S6), for usual mode of commuting to and from school, frequency of ACS, perceived barriers to ACS, BPNS related to ACS, motivation for ACS, and all PA outcomes, except for amotivation. Girls in the intervention group showed greater increases in amotivation for ACS compared with their counterparts in the control group (mean difference: 0.54; *p* = 0.018) (Figure S2).

### Per-protocol analysis

The per-protocol analysis, which represents the sensitivity analysis, is presented in the Supplementary Material (Tables S4–S7, Figures S7–S13). Regarding the attrition and session attendance (Fig. 1), for the per-protocol analyses of ACS outcomes, participants were excluded due to insufficient intervention attendance (< 75%; intervention group only, *n* = 30) or incomplete pre- or post-intervention ACS data (intervention: *n* = 45; control: *n* = 50). Similarly, for PA outcomes, exclusions were due to insufficient session attendance (intervention group only, *n* = 30) or incomplete accelerometry data at baseline or post-intervention (intervention: *n* = 72; control: *n* = 123). Overall, the results were similar to those obtained in the intention-to-treat approach, except for the motivation for ACS, where participants in the intervention group showed higher external motivation (mean difference = 0.38, *p* = 0.037; d = 0.38) and amotivation for ACS (mean difference = 0.45, *p* = 0.030; d = 0.39) compared to the control group after the school-based cycling intervention (Table S6). On the other hand, the moderation analyses revealed more differences compared to the ITT analysis. Girls in the intervention group showed an increase in their perceived environmental/safety barriers to ACS (mean difference = 0.40; *p* = 0.029) and external motivation (mean difference = 0.47; *p* = 0.047) compared to girls in the control group (Figure S9). Moreover, participants in the high-SES group showed an increase in both perceived environmental/safety (mean difference = 0.47; *p* = 0.010) and planning/psychosocial barriers to ACS (mean difference = 0.40; *p* = 0.028), and in their amotivation for ACS compared to their peers in the control group (mean difference = 0.54; *p* = 0.047) (Figure S10).

## Discussion

The main findings indicate no differential changes between groups in the usual mode or in the frequency of ACS to school from baseline to follow-up. Similarly, no group-by-time effects were observed for most ACS-related psychosocial outcomes. However, perceived environmental/safety barriers to ACS increased in the intervention group compared with the control group after the program. Device-measured sedentary time and PA also did not differ between groups across daily segments. Moreover, moderation analyses revealed subgroup-specific responses to the intervention. Girls allocated to the intervention showed greater increases in amotivation toward ACS compared with their peers in the control group. Per-protocol analyses were broadly consistent with the intention-to-treat findings, although additional differences emerged, indicating higher external motivation and amotivation for ACS in the intervention group. Regarding subgroup patterns, girls reported higher perceived environmental/safety barriers and external motivation for ACS, while adolescents from higher socioeconomic backgrounds showed higher environmental/safety and planning/psychosocial barriers, as well as higher amotivation, compared with their peers in the control group. Thus, these findings suggest that the intervention influenced how adolescents perceived cycling and commuting, even in the absence of behavioral change.

No intervention effect on the mode of commuting or on the frequency of ACS was detected, which is consistent with other school-based cycling programs [46, 47]. However, other studies have reported meaningful shifts when family engagement was incorporated, a component not feasible in the present study, suggesting that parental support can catalyze behavior change [48–50]. Yet evidence remains mixed, as other trials have found no effect even with parental involvement [20, 51]. Another factor that may explain the lack of change in the current study in the mode of commuting is the relatively long distance to school, with participants living on average more than three kilometers away. Distance is widely recognized as the main barrier to ACS [52]. Although this exceeds the commonly suggested walking threshold for adolescents (≈ 1.3 km) [53], it remains below the cycling threshold reported in the only Spanish study to date (≈ 5 km) [54]. Beyond these structural constraints (e.g., limited family engagement and relatively long home–school distance), the psychosocial findings provide a plausible explanatory pathway for the lack of behavior change in the present intervention.

The school-based cycling intervention was associated with a significant increase in perceived environmental/safety barriers, suggesting a meaningful shift in how adolescents understand the influence of the environment and safety of cycling to school. A plausible explanation is that many participants cycled in urban traffic for the first time and became more attuned to challenges such as intersections, roundabouts, and sharing lanes with motor vehicles. Rather than being a mere side effect of the program’s short duration, this increase in perceived barriers may help explain the null behavioral effects. Heightened hazard salience and perceived constraints could have reduced the perceived feasibility and attractiveness of cycling for commuting to school, thereby counteracting uptake. These findings suggest that the intervention primarily influenced how adolescents perceived cycling, by increasing awareness of risks and practical challenges, rather than changing their actual mode of commuting to school. This interpretation is further supported by the fact that nearly half of the students did not even have a bicycle in good condition, suggesting limited regular cycling experience and reinforcing the distinction between cycling skills practiced in controlled settings and cycling in real-world traffic conditions. This aligns with students’ opinions from the pilot feasibility study, in which the urban circuit session was the one they liked the most, although cycling through the roundabout was reported as difficult [55]. Furthermore, as it was not a regular behavior for them, novelty should be acting as a boost to the autonomous motivation of adolescents. Moreover, BPNS related to ACS and motivation for ACS showed no between-group differences. The current intervention was too short to derive in any positive change; however, it was the most feasible duration for Physical Education teachers. Other trials have similarly found minimal impact on motivational correlates of ACS when using brief educational approaches [56, 57]. By contrast, longer interventions, such as supervised “bike-bus” programs where adults regularly guided students cycling to school, have achieved improvements in psychosocial outcomes [58]. Such multi-faceted, high-support strategies appear more effective than curriculum-only interventions [18], which may help explain why the present low-dose program did not produce significant changes in commuting outcomes. Thus, future interventions should combine longer cycling skills training with gender-responsive and context-tailored strategies (e.g., autonomy-supportive delivery, family/school engagement, and route-safety measures) to address group-specific barriers.

From a practical view, beyond family involvement, peer influence may represent an important yet underexplored pathway for promoting ACS during adolescence. Observational evidence suggests that friends’ behaviors and social support are associated with ACS and that transport choices cluster within friendship networks, particularly for cycling [59, 60]. Moreover, peer-led school programs have shown that adolescents can successfully deliver activity-related interventions within the school context, supporting the feasibility of peer-based delivery strategies [61]. For cycling-specific initiatives, group-based approaches such as “bicycle trains” (i.e., cycling with others under supervision) have increased cycling-to-school in experimental studies, suggesting that structured group rides may strengthen social support and norms around active transport [25]. In future school cycling programs, parental involvement could be incorporated through brief, low-burden components (e.g., informational sessions, safety reassurance, coordinated permissions, and route planning) to address safety concerns without undermining adolescents’ autonomy. Regarding gender, prior research consistently shows lower cycling-related engagement among girls [42, 62]. Evidence from school-based ACS interventions also suggests that effects may differ by gender, with some programs reporting more favorable cycling changes among boys than girls [42]. Our findings (greater increases in perceived barriers and amotivation among girls and higher-SES students), this supports the need for gender-responsive, rather than fully gender-segregated, strategies that explicitly address safety perceptions, social norms, and autonomy-supportive delivery [63]. From a policy perspective, our results reinforce that school-based cycling programs may be insufficient unless embedded in broader initiatives considering built environmental factors (e.g., safer school routes, traffic calming) and multisectoral collaborations and cross-sector coordination between schools, municipalities, and families) [64].

The per-protocol analyses were consistent with ITT findings. However, additional differences emerged after the intervention, with increases in external motivation in the intervention group compared to the control group. These findings could be explained by the limited duration of the program, which was likely insufficient to foster more self-determined forms of motivation, such as identified or intrinsic regulation [65, 66]. Evidence indicates that longer, more comprehensive, and need-supportive interventions, often involving family or peers, are more effective at enhancing autonomous motivation, whereas short, skills-focused curricula tend to yield modest or null behavior change unless accompanied by stronger and sustained support [65, 66]. The intervention also increased participants’ amotivation toward ACS. While participants acquired cycling skills and different knowledges on cycling safety, traffic rules and equipment checks, the challenges of riding in real urban traffic may have heightened perceptions of difficulty and risk. Prior research shows that when perceived barriers are salient and not fully addressed, adolescents may feel less capable or view the behavior as beyond their control, which is a known antecedent of amotivation [23].

In moderation analyses by sex, girls allocated to the intervention exhibited an increase in amotivation toward ACS compared to their peers in the control group. Furthermore, in the per-protocol moderation analyses, girls in the intervention group reported higher perceived environmental/safety barriers and higher external regulation compared with girls in the control group. These results could be explained by the fact that girls perceive multifactorial barriers, including traffic exposure, intersections and roundabouts, appearance, and peer norms, which may reinforce the perception that cycling is effortful or unsafe, thereby increasing amotivation [67, 68]. This greater sensitivity to safety and environmental concerns may also reflect broader gendered social and cultural factors that shape perceived safety and the social acceptability of cycling and independent mobility during adolescence. Evidence from observational research shows that cycling (vs. walking) is often perceived as less safe and receives less parental/peer encouragement, which may disproportionately affect girls’ willingness to cycle to school [69, 70]. Skills-focused programs can increase hazard salience in real-world cycling (e.g., intersections, roundabouts, and sharing carriageways), especially for girls, who typically report greater safety concerns and lower perceived cycling competence [71–73]. Furthermore, the per-protocol analyses showed that adolescents with high SES in the intervention group reported higher perceived environmental/safety and planning/psychosocial barriers, as well as higher amotivation, than their peers in the control group. In higher-affluence settings, easy access to motorized transport and convenience norms can reinforce the perception that cycling is unnecessary or effortful once risks become more visible [74]. This interpretation is consistent with Spanish evidence showing that higher socioeconomic indicators are inversely associated with ACS in adolescents, likely reflecting higher motorized access and convenience-oriented routines [62]. In addition, adolescents’ mobility aspirations may be shaped by car-oriented norms in more affluent contexts, which can further reduce the perceived utility of cycling for school travel when constraints are salient [75]. These patterns suggest that the program may have heightened awareness of traffic risks and shifted the motivational climate toward more controlled forms without parallel improvements in contextual conditions (e.g., safer routes, traffic calming, or parental and school-level support) [22, 46]. These findings suggest that brief, skills-based cycling programs with sessions delivered in real-traffic contexts may increase adolescents’ awareness of environmental/safety and planning/psychosocial constraints, without being sufficient to internalize and develop the skills needed to overcome perceived risks. This may help explain the observed increases in perceived barriers, external regulation, and amotivation, among the intervention group, particularly among girls and adolescents attending higher-SES schools, and indicates that future interventions should combine cycling skills training with gender-responsive and context-tailored strategies (e.g., autonomy-supportive delivery, family/school engagement, and route-safety measures) to address group-specific barriers.

Finally, the cycling intervention did not produce detectable changes in either ITT or per-protocol analyses for device-measured sedentary time or PA, which aligns with previous interventions promoting ACS [21, 28]. From a behavioral perspective, this finding is coherent with the absence of change in the mode of commuting to school, as short-term educational or skills-based interventions are unlikely to modify total daily activity unless they result in sustained changes in habitual commuting behavior [17]. Future studies focusing on assessing ACS-related PA should consider combining accelerometry with Global Positioning System (GPS) data to more precisely capture activity changes occurring during ACS and in adjacent time periods beyond the school context, and to better isolate intervention-related effects on domain-specific PA [76].

The current study is not without limitations. The usual mode and the frequency of walking and cycling trips were assessed only during the commuting to and from school, leaving unknown whether the intervention influenced other trip contexts (e.g., visiting friends or traveling to recreational areas). Follow-up was limited to the immediate post-intervention window, which may not capture longer-term effects. The Bikeability program was delivered in only four lessons, limiting opportunities to practice cycling in real urban traffic. Another limitation relates to adherence, as reflected in the drop-out between the ITT and per-protocol analyses. This loss was mainly due to COVID-19, which prevented post-test data collection for some participants with respect to accelerometry. Nevertheless, the absence of effects in both approaches suggests that the intervention’s limitations (e.g., short duration, lack of parental involvement) were fundamental rather than merely a dilution caused by non-compliers. Although accelerometry provided objective estimates across daily segments, the absence of concurrent GPS meant that activity could not be attributed specifically to commuting, so any commute-related increase may have been overlooked. In addition, even though no changes were observed in the number of cycling trips from pre- to post-intervention, it is important to acknowledge the limitations of accelerometers in capturing PA during cycling [77]. Finally, qualitative methods (e.g., focus groups) were not used, which could have provided valuable insights into feasibility, acceptability, perceived barriers, and helped explain the ACS-related psychosocial outcomes.

## Conclusions

The present study provides important lessons for the design of future school-based cycling initiatives. Although the PACO school-based cycling intervention did not produce significant changes in the mode or weekly frequency of ACS, ACS-related psychosocial outcomes, or device-measured sedentary time and PA, it yielded meaningful insights into why behavior change did not occur. Specifically, the intervention was associated with higher perceived environmental/safety barriers and greater amotivation for ACS, particularly among girls. Moreover, in the per-protocol analysis, participants who adhered to the intervention showed increases in external regulation and amotivation, with effects especially pronounced among girls and adolescents from higher socioeconomic backgrounds, who also reported greater perceived planning/psychosocial barriers post-intervention. These findings suggest that cycling education and skills training delivered in isolation may be insufficient, and, in some contexts, may even heighten perceived constraints, if not accompanied by supportive built environmental and social conditions. Future cycling initiatives should combine sustained skills training with improvements in the built environment, such as safe routes, protected infrastructure, and supervision (e.g., protected links, safer crossings, secure bike parking), alongside parental engagement and clear school arrival and parking procedures. Delivering such programs in an autonomy-supportive manner may strengthen adolescents’ perceptions of safety, confidence, and willingness to cycle.

## Supplementary Information

Supplementary Material 1.

## Data Availability

The datasets used and/or analyzed during the current study are available from the corresponding author on reasonable request.

## Abbreviations

ACS: Active commuting to/from school
PA: Physical activity
LPA: Light physical activity
MVPA: Moderate-to-vigorous physical activity
BPNS: Basic psychological needs
GPS: Global positioning system
SES: Socioeconomic status
SD: Standard deviation
ANCOVA: Analysis of covariance
ITT: Intention to treat

## References

[1] Poitras VJ, Gray CE, Borghese MM, Carson V, Chaput J-P, Janssen I, et al. Systematic review of the relationships between objectively measured physical activity and health indicators in school-aged children and youth. Appl Physiol Nutr Metab. 2016;41:S197–239. 10.1139/apnm-2015-0663. 27306431 10.1139/apnm-2015-0663

[2] Rodriguez-Ayllon M, Cadenas-Sánchez C, Estévez-López F, Muñoz NE, Mora-Gonzalez J, Migueles JH, et al. Role of Physical Activity and Sedentary Behavior in the Mental Health of Preschoolers, Children and Adolescents: A Systematic Review and Meta-Analysis. Sports Med. 2019;49:1383–410. 10.1007/s40279-019-01099-5. 30993594 10.1007/s40279-019-01099-5

[3] Aubert S, Barnes JD, Demchenko I, Hawthorne M, Abdeta C, Abi Nader P, et al. Global Matrix 4.0 Physical Activity Report Card Grades for Children and Adolescents: Results and Analyses From 57 Countries. J Phys Act Health. 2022;19:700–28. 10.1123/jpah.2022-0456. 36280233 10.1123/jpah.2022-0456

[4] Wargama IMDS, Rahayu T, Priyono B, Mukarromah SB, Pramono H, Setyawati H, et al. What is the relationship between socioeconomics and physical activity? Literature review. Retos. 2024;61:148–55. 10.47197/retos.v61.109628.

[5] Guthold R, Stevens GA, Riley LM, Bull FC. Global trends in insufficient physical activity among adolescents: a pooled analysis of 298 population-based surveys with 1·6 million participants. Lancet Child Adolesc Health. 2020;4:23–35. 10.1016/S2352-4642(19)30323-2. 31761562 10.1016/S2352-4642(19)30323-2 PMC6919336

[6] Cook S, Stevenson L, Aldred R, Kendall M, Cohen T. More than walking and cycling: What is ‘active travel’? Transp Policy (Oxf). 2022;126:151–61. 10.1016/j.tranpol.2022.07.015.

[7] Campos-Garzón P, Sevil-Serrano J, García-Hermoso A, Chillón P, Barranco-Ruiz Y. Contribution of active commuting to and from school to device-measured physical activity levels in young people: A systematic review and meta-analysis. Scand J Med Sci Sports. John Wiley and Sons Inc; 2023: 2110–24. 10.1111/sms.14450. 10.1111/sms.14450 37497601

[8] Larouche R, Saunders TJ, Faulkner GEJ, Colley R, Tremblay M. Associations between active school transport and physical activity, body composition, and cardiovascular fitness: A systematic review of 68 studies. J Phys Act Health. 2014;11:206–27. 10.1123/jpah.2011-0345. 23250273 10.1123/jpah.2011-0345

[9] Ding D, Luo M, Infante MFP, Gunn L, Salvo D, Zapata-Diomedi B, et al. The co-benefits of active travel interventions beyond physical activity: a systematic review. Lancet Planet Health Elsevier B V. 2024;e790–803. 10.1016/S2542-5196(24)00201-8. 10.1016/S2542-5196(24)00201-8 39393380

[10] Meron D, Rissel C, Reinten-Reynolds T, Hardy LL. Changes in active travel of school children from 2004 to 2010 in New South Wales, Australia. Prev Med (Baltim). 2011;53:408–10. 10.1016/j.ypmed.2011.09.017. 10.1016/j.ypmed.2011.09.017 22020058

[11] MCDONALD N. Active Transportation to SchoolTrends, Among US, Schoolchildren. 1969–2001. Am J Prev Med. 2007;32:509–16. 10.1016/j.amepre.2007.02.022. 10.1016/j.amepre.2007.02.022 17533067

[12] Pavelka J, Sigmundová D, Hamřík Z, Kalman M, Sigmund E, Mathisen F. Trends in Active Commuting to School among Czech Schoolchildren from 2006 to 2014. Cent Eur J Public Health. 2017;25:S21–5. 10.21101/cejph.a5095. 28752743 10.21101/cejph.a5095

[13] Gálvez-Fernández P, Herrador‐Colmenero M, Esteban‐Cornejo I, Castro‐Piñero J, Molina‐García J, Queralt A, et al. Active commuting to school among 36,781 Spanish children and adolescents: A temporal trend study. Scand J Med Sci Sports. 2021;31:914–24. 10.1111/sms.13917. 33423302 10.1111/sms.13917

[14] Herrador-Colmenero M, Escabias M, Ortega FB, McDonald NC, Chillon P. Mode of Commuting TO and FROM School: A Similar or Different Pattern? Sustainability. Univ Granada, Fac Sport Sci, Dept Phys Educ & Sport, PROFITH PROmoting FITness & Hlth Phys Act Res Grp, Granada 18011, Spain; 2019;11. 10.3390/su11041026.

[15] Ramírez-Vélez R, García-Hermoso A, Agostinis-Sobrinho C, Mota J, Santos R, Correa-Bautista JE, et al. Cycling to School and Body Composition, Physical Fitness, and Metabolic Syndrome in Children and Adolescents. J Pediatr United States. 2017;188:57–63. 10.1016/j.jpeds.2017.05.065. 10.1016/j.jpeds.2017.05.065 28651798

[16] Larouche R, Mammen G, Rowe DA, Faulkner G. Effectiveness of active school transport interventions: a systematic review and update. BMC Public Health. 2018;18:206. 10.1186/s12889-017-5005-1. 29390988 10.1186/s12889-017-5005-1 PMC5796594

[17] Villa-González E, Barranco-Ruiz Y, Evenson KR, Chillón P, Villa-Gonzalez E, Barranco-Ruiz Y et al. Systematic review of interventions for promoting active school transport. Prev Med (Baltim) [Internet]. Univ Granada, PROFITH PROmoting FITness & Hlth Phys Act Res Grp, Fac Sport Sci, Dept Phys Educ & Sport, Ctra Alfacar SN, Granada 18070, Spain: Elsevier; 2018;111:115–34. 10.1016/j.ypmed.2018.02.010. 10.1016/j.ypmed.2018.02.010 29496615

[18] Schönbach DMI, Altenburg TM, Marques A, Chinapaw MJM, Demetriou Y. Strategies and effects of school-based interventions to promote active school transportation by bicycle among children and adolescents: a systematic review. Int J Behav Nutr Phys Act Engl. 2020;17:138. 10.1186/s12966-020-01035-1. 10.1186/s12966-020-01035-1 PMC7661215 33183331

[19] Xu F, Ware RS, Leslie E, Tse LA, Wang Z, Li J, et al. Effectiveness of a Randomized Controlled Lifestyle Intervention to Prevent Obesity among Chinese Primary School Students: CLICK-Obesity Study. PLoS ONE. 2015;10:e0141421. 10.1371/journal.pone.0141421. 26510135 10.1371/journal.pone.0141421 PMC4625022

[20] Villa-Gonzalez E, Ruiz JR, Ward DS, Chillon P, Villa-González E, Ruiz JR, et al. Effectiveness of an active commuting school-based intervention at 6-month follow-up. Eur J Public Health Engl. 2016;26:272–6. 10.1093/eurpub/ckv208. 10.1093/eurpub/ckv208 26578663

[21] Østergaard L, Støckel JT, Andersen LB. Effectiveness and implementation of interventions to increase commuter cycling to school: a quasi-experimental study. BMC Public Health Engl. 2015;15:1199. 10.1186/s12889-015-2536-1. 10.1186/s12889-015-2536-1 PMC4665862 26619996

[22] Buttazzoni A, Nelson Ferguson K, Gilliland J. Barriers to and facilitators of active travel from the youth perspective: A qualitative meta-synthesis. SSM Popul Health. 2023;22:101369. 10.1016/j.ssmph.2023.101369. 36909930 10.1016/j.ssmph.2023.101369 PMC9996358

[23] Ntoumanis N, Ng JYY, Prestwich A, Quested E, Hancox JE, Thøgersen-Ntoumani C, et al. A meta-analysis of self-determination theory-informed intervention studies in the health domain: effects on motivation, health behavior, physical, and psychological health. Health Psychol Rev. 2021;15:214–44. 10.1080/17437199.2020.1718529. 31983293 10.1080/17437199.2020.1718529

[24] Niven AG, Markland D. Using self-determination theory to understand motivation for walking: Instrument development and model testing using Bayesian structural equation modelling. Psychol Sport Exerc. 2016;23:90–100. 10.1016/j.psychsport.2015.11.004.

[25] Mendoza JA, Haaland W, Jacobs M, Abbey-Lambertz M, Miller J, Salls D, et al. Bicycle Trains, Cycling, and Physical Activity: A Pilot Cluster RCT. Am J Prev Med. 2017;53:481–9. 10.1016/j.amepre.2017.05.001. 28668251 10.1016/j.amepre.2017.05.001 PMC5894119

[26] Mendoza JA, Watson K, Baranowski T, Nicklas TA, Uscanga DK, Hanfling MJ. The Walking School Bus and Children’s Physical Activity: A Pilot Cluster Randomized Controlled Trial. Pediatrics. Baylor Univ, USDA ARS, Childrens Nutr Res Ctr, Coll Med, Houston, TX 77030 USA; 2011;128:E537–44. 10.1542/peds.2010-3486. 10.1542/peds.2010-3486 PMC3164094 21859920

[27] Vanwolleghem G, D’Haese S, Van Dyck D, De Bourdeaudhuij I, Cardon G. Feasibility and effectiveness of drop-off spots to promote walking to school. Int J Behav Nutr Phys Activity. 2014;11:136. 10.1186/s12966-014-0136-6. 10.1186/s12966-014-0136-6 PMC4220063 25346220

[28] Sayers SP, LeMaster JW, Thomas IM, Petroski GF, Ge B. A Walking School Bus program: impact on physical activity in elementary school children in Columbia, Missouri. Am J Prev Med Neth. 2012;43:S384–9. 10.1016/j.amepre.2012.07.009. 10.1016/j.amepre.2012.07.009 23079270

[29] McMinn D, Rowe DA, Murtagh S, Nelson NM. The effect of a school-based active commuting intervention on children’s commuting physical activity and daily physical activity. Prev Med (Baltim) United States. 2012;54:316–8. 10.1016/j.ypmed.2012.02.013. 10.1016/j.ypmed.2012.02.013 22405706

[30] Goodman A, van Sluijs E, Ogilvie D. ⁎A28 Impact of ‘Bikeability’, a national cycle training scheme for children in England. J Transp Health. 2015;2:S19. 10.1016/j.jth.2015.04.516.

[31] Chillon P, Galvez-Fernandez P, Huertas-Delgado FJ, Herrador-Colmenero M, Barranco-Ruiz Y, Villa-Gonzalez E, et al. A School-Based Randomized Controlled Trial to Promote Cycling to School in Adolescents: The PACO Study. Int J Environ Res Public Health. 2021;18. 10.3390/ijerph18042066. 10.3390/ijerph18042066 PMC7923771 33672550

[32] Segura-Díaz J, Rojas-Jiménez Á, Barranco-Ruiz Y, Murillo-Pardo B, Saucedo-Araujo R, Aranda-Balboa M, et al. Feasibility and Reliability of a Questionnaire to Assess the Mode, Frequency, Distance and Time of Commuting to and from School: The PACO Study. Int J Environ Res Public Health. 2020;17:5039. 10.3390/ijerph17145039. 32668796 10.3390/ijerph17145039 PMC7399968

[33] Chillón P, Herrador-Colmenero M, Migueles JH, Cabanas-Sánchez V, Fernández-Santos JR, Veiga ÓL, et al. Convergent validation of a questionnaire to assess the mode and frequency of commuting to and from school. Scand J Public Health Univ Granada. 2017;45:612–20. 10.1177/1403494817718905. Dept Phys Educ & Sport, Fac Sport Sci, PROFITH Promoting FITness & Hlth Phys Act Res Grp, Granada, Spain, Sweden. 10.1177/1403494817718905 30747037

[34] Burgueño R, González-Cutre D, Sevil-Serrano J, Herrador-Colmenero M, Segura-Díaz JM, Medina-Casaubón J, et al. Validation of the Basic Psychological Need Satisfaction in Active Commuting to and from School (BPNS-ACS) Scale in Spanish young people. J Transp Health. 2020;16:100825. 10.1016/j.jth.2020.100825.

[35] Burgueño R, González-Cutre D, Sevil-Serrano J, Herrador-Colmenero M, Segura-Díaz JM, Medina-Casaubón J, et al. Understanding the motivational processes involved in adolescents’ active commuting behaviour: Development and validation of the Behavioural Regulation in Active Commuting to and from School (BR-ACS) Questionnaire. Transp Res Part F Traffic Psychol Behav. 2019;62:615–25. 10.1016/j.trf.2019.02.016.

[36] Migueles JH, Rowlands AV, Huber F, Sabia S, van Hees VT. GGIR: A Research Community–Driven Open Source R Package for Generating Physical Activity and Sleep Outcomes From Multi-Day Raw Accelerometer Data. J Meas Phys Behav. 2019;2:188–96. 10.1123/jmpb.2018-0063.

[37] van Hees VT, Renström F, Wright A, Gradmark A, Catt M, Chen KY, et al. Estimation of Daily Energy Expenditure in Pregnant and Non-Pregnant Women Using a Wrist-Worn Tri-Axial Accelerometer. PLoS ONE. 2011;6:e22922. 10.1371/journal.pone.0022922. 21829556 10.1371/journal.pone.0022922 PMC3146494

[38] Neishabouri A, Nguyen J, Samuelsson J, Guthrie T, Biggs M, Wyatt J, et al. Quantification of acceleration as activity counts in ActiGraph wearable. Sci Rep. 2022;12:11958. 10.1038/s41598-022-16003-x. 35831446 10.1038/s41598-022-16003-x PMC9279376

[39] Evenson KR, Catellier DJ, Gill K, Ondrak KS, McMurray RG. Calibration of two objective measures of physical activity for children. J Sports Sci. 2008;26:1557–65. 10.1080/02640410802334196. 18949660 10.1080/02640410802334196

[40] Currie C, Molcho M, Boyce W, Holstein B, Torsheim T, Richter M. Researching health inequalities in adolescents: The development of the Health Behaviour in School-Aged Children (HBSC) Family Affluence Scale. Soc Sci Med. 2008;66:1429–36. 10.1016/j.socscimed.2007.11.024. 18179852 10.1016/j.socscimed.2007.11.024

[41] Coffman CJ, Edelman D, Woolson RF. To condition or not condition? Analysing ‘change’ in longitudinal randomised controlled trials. BMJ Open. 2016;6:e013096. 10.1136/bmjopen-2016-013096. 28039292 10.1136/bmjopen-2016-013096 PMC5223669

[42] Villa-González E, Ruiz JR, Mendoza JA, Chillón P. Effects of a school-based intervention on active commuting to school and health-related fitness. BMC Public Health 2017 17:1. BioMed Central; 2017;17:20. 10.1186/S12889-016-3934-8. Cited 2025 Dec 28. 10.1186/s12889-016-3934-8 PMC5216538 28056914

[43] White IR, Horton NJ, Carpenter J, statistics r. i. m. a. s., Pocock SJ. Strategy for intention to treat analysis in randomised trials with missing outcome data. BMJ. 2011;342:d40–d40. 10.1136/bmj.d40. 10.1136/bmj.d40 PMC3230114 21300711

[44] Nakagawa S, Cuthill IC. Effect size, confidence interval and statistical significance: a practical guide for biologists. Biol Rev. 2007;82:591–605. 10.1111/j.1469-185X.2007.00027.x. 17944619 10.1111/j.1469-185X.2007.00027.x

[45] Benjamini Y, Hochberg Y. Controlling the False Discovery Rate: A Practical and Powerful Approach to Multiple Testing. J R Stat Soc Ser B Stat Methodol. 1995;57:289–300. 10.1111/j.2517-6161.1995.tb02031.x.

[46] Goodman A, van Sluijs EMF, Ogilvie D. Impact of offering cycle training in schools upon cycling behaviour: a natural experimental study. Int J Behav Nutr Phys Activity. 2016;13:34. 10.1186/s12966-016-0356-z. 10.1186/s12966-016-0356-z PMC4784314 26956383

[47] Coombes E, Jones A. Gamification of active travel to school: A pilot evaluation of the Beat the Street physical activity intervention. Health Place. 2016;39:62–9. 10.1016/j.healthplace.2016.03.001. 26974232 10.1016/j.healthplace.2016.03.001 PMC5405045

[48] Hatfield J, Boufous S, Eveston T. An evaluation of the effects of an innovative school-based cycling education program on safety and participation. Accid Anal Prev. 2019;127:52–60. 10.1016/j.aap.2019.02.021. 30831538 10.1016/j.aap.2019.02.021

[49] Jones P. The Impact of Cycle Skills Training on Skills, Confidence, Attitudes and Rates of Cycling [Master’s Thesis]. [Waterford, Ireland]: Waterford Institute of Technology; 2017.

[50] Montenegro S. Evaluating Cycle Kids: A Bicycling and Nutrition Health Promotion Curriculum Delivered as a Component of School Based Physical Education [Master’s Thesis]. [Boston, MA, USA]: Boston University; 2015.

[51] Ducheyne F, De Bourdeaudhuij I, Lenoir M, Cardon G. Effects of a cycle training course on children’s cycling skills and levels of cycling to school. Accid Anal Prev. 2014;67:49–60. 10.1016/j.aap.2014.01.023. 24607594 10.1016/j.aap.2014.01.023

[52] Panter JR, Jones AP, van Sluijs EM. Environmental determinants of active travel in youth: A review and framework for future research. Int J Behav Nutr Phys Activity. 2008;5:34. 10.1186/1479-5868-5-34. 10.1186/1479-5868-5-34 PMC2483993 18573196

[53] Rodríguez-López C, Salas-Fariña ZM, Villa-González E, Borges-Cosic M, Herrador-Colmenero M, Medina-Casaubón J, et al. The Threshold Distance Associated With Walking From Home to School. Health Educ Behav. 2017;44:857–66. 10.1177/1090198116688429. 28178850 10.1177/1090198116688429

[54] Chillón P, Molina-García J, Castillo I, Queralt A. What distance do university students walk and bike daily to class in Spain. J Transp Health [Internet] Elsevier. 2016;3:315–20. 10.1016/J.JTH.2016.06.001. Cited 2025 Sep 22.

[55] Aranda-Balboa MJ, Huertas-Delgado FJ, Gálvez-Fernández P, Saucedo-Araujo R, Molina-Soberanes D, Campos-Garzón P et al. The Effect of a School-Based Intervention on Children’s Cycling Knowledge, Mode of Commuting and Perceived Barriers: A Randomized Controlled Trial. International Journal of Environmental Research and Public Health 2022, Vol 19, Page 9626. Multidisciplinary Digital Publishing Institute; 2022;19:9626. 10.3390/IJERPH19159626. Cited 2025 Oct 25. 10.3390/ijerph19159626 PMC9367827 35954982

[56] Christiansen LB, Toftager M, Ersbøll AK, Troelsen J. Effects of a Danish multicomponent physical activity intervention on active school transport. J Transp Health. 2014;1:174–81. 10.1016/j.jth.2014.05.002.

[57] Gutierrez CM, Slagle D, Figueras K, Anon A, Huggins AC, Hotz G. Crossing guard presence: Impact on active transportation and injury prevention. J Transp Health. 2014;1:116–23. 10.1016/j.jth.2014.01.005.

[58] Huang C, Dannenberg AL, Haaland W, Mendoza JA. Changes in Self-Efficacy and Outcome Expectations From Child Participation in Bicycle Trains for Commuting to and From School. Health Education & Behavior. SAGE PublicationsSage CA: Los Angeles, CA; 2018; 45:748–55. 10.1177/1090198118769346. Cited 2025 Oct 17. 10.1177/1090198118769346 PMC6598703 29631444

[59] Panter JR, Jones AP, Van Sluijs EMF, Griffin SJ. Attitudes, social support and environmental perceptions as predictors of active commuting behaviour in school children. J Epidemiol Community Health. 1978;64:41–8. 10.1136/JECH.2009.086918. Cited 2025 Dec 28. 10.1136/jech.2009.086918 PMC3703574 19465403

[60] Long J, Harré N, Atkinson QD. Social clustering in high school transport choices. J Environ Psychol. 2015;41:155–65. 10.1016/j.jenvp.2015.01.001.

[61] Carlin A, Murphy MH, Nevill A, Gallagher AM. Effects of a peer-led Walking In ScHools intervention (the WISH study) on physical activity levels of adolescent girls: a cluster randomised pilot study. Trials 2018 19:1. BioMed Central; 2018;19:31–. 10.1186/S13063-017-2415-4. Cited 2025 Dec 28. 10.1186/s13063-017-2415-4 PMC5765605 29325578

[62] Chillón P, Ortega FB, Ruiz JR, Veidebaum T, Oja L, Mäestu J, et al. Active commuting to school in children and adolescents: An opportunity to increase physical activity and fitness. Scand J Public Health. 2010;38:873–9. 10.1177/1403494810384427. 20855356 10.1177/1403494810384427

[63] Millet GP, Hill J, Marzi I, Emmerling S, Demetriou Y, Bucksch J et al. Interventions Aiming to Promote Active Commuting in Children and Adolescents: An Evaluation From a Sex/Gender Perspective. Front Sports Act Living. Frontiers; 2020;2:590857. 10.3389/FSPOR.2020.590857. Cited 2025 Dec 28. 10.3389/fspor.2020.590857 PMC7739596 33345167

[64] Zukowska J, Gobis A, Krajewski P, Morawiak A, Okraszewska R, Woods CB, et al. Which transport policies increase physical activity of the whole of society? A systematic review. J Transp Health. 2022;27:101488. 10.1016/j.jth.2022.101488.

[65] Sweet SN, Fortier MS, Blanchard CM. Investigating Motivational Regulations and Physical Activity Over 25 Weeks. J Phys Act Health. 2014;11:1052–6. 10.1123/jpah.2012-0057. 23799262 10.1123/jpah.2012-0057

[66] Teixeira PJ, Carraça EV, Markland D, Silva MN, Ryan RM. Exercise, physical activity, and self-determination theory: A systematic review. Int J Behav Nutr Phys Activity. 2012;9:78. 10.1186/1479-5868-9-78. 10.1186/1479-5868-9-78 PMC3441783 22726453

[67] Aranda-Balboa MJ, Chillón P, Saucedo-Araujo RG, Molina-García J, Huertas-Delgado FJ. Children and Parental Barriers to Active Commuting to School: A Comparison Study. Int J Environ Res Public Health. 2021;18:2504. 10.3390/ijerph18052504. 33802516 10.3390/ijerph18052504 PMC7967632

[68] Pinilla-Quintana I, Martin-Moraleda E, Romero-Blanco C, Hernández-Martínez A, Švátora K, Martínez-Romero MT et al. Differences between adolescents’ and their parents’ perceived benefits and barriers to actively commute to school: The PACO y PACA project. J Transp Health. Elsevier Ltd; 2024. 10.1016/j.jth.2024.101889.

[69] Mandic S, Hopkins D, García Bengoechea E, Flaherty C, Williams J, Sloane L, et al. Adolescents’ perceptions of cycling versus walking to school: Understanding the New Zealand context. J Transp Health [Internet] Elsevier. 2017;4:294–304. 10.1016/J.JTH.2016.10.007. [cited 2025 Dec 29];.

[70] Leslie E, Kremer P, Toumbourou JW, Williams JW. Gender differences in personal, social and environmental influences on active travel to and from school for Australian adolescents. J Sci Med Sport [Internet] J Sci Med Sport. 2010;13:597–601. 10.1016/j.jsams.2010.04.004. [cited 2025 Dec 29]. 20594909 10.1016/j.jsams.2010.04.004

[71] Teyhan A, Cornish R, Boyd A, Sissons Joshi M, Macleod J. The impact of cycle proficiency training on cycle-related behaviours and accidents in adolescence: findings from ALSPAC, a UK longitudinal cohort. BMC Public Health. 2016;16:469. 10.1186/s12889-016-3138-2. 27276877 10.1186/s12889-016-3138-2 PMC4899925

[72] Graystone M, Mitra R, Hess PM. Gendered perceptions of cycling safety and on-street bicycle infrastructure: Bridging the gap. Transp Res D Transp Environ. 2022;105:103237. 10.1016/j.trd.2022.103237.

[73] Higgins R, Ahern A. Students’ and Parents’ Perceptions of Barriers to Cycling to School—An Analysis by Gender. Sustainability. 2021;13:13213. 10.3390/su132313213.

[74] Van Sluijs EMF, Ekelund U, Hallal PC, Hansen BH, Panter J, Salmon J, et al. Family Car Ownership: Driving Inactivity in Young People? Cross-Sectional and Longitudinal Analyses in the International Children’s Accelerometry Database. J Phys Act Health. 2024;21:1391–400. 10.1123/jpah.2024-0044. 39424287 10.1123/jpah.2024-0044

[75] Hopkins D, García Bengoechea E, Mandic S. Adolescents and their aspirations for private car-based transport. Transp (Amst). 2021;48:67–93. 10.1007/s11116-019-10044-4.

[76] Campos-Garzón P, Saucedo-Araujo RG, Sevil-Serrano J, Migueles JH, Barranco-Ruiz Y, Chillón P. A systematic review in device-measured physical activity during active commuting to/from school: practical considerations to assess when, where, and how much it occurs. Transp Rev. 2023;1–26. 10.1080/01441647.2023.2175276.

[77] Tarp J, Andersen LB, Østergaard L. Quantification of Underestimation of Physical Activity During Cycling to School When Using Accelerometry. J Phys Act Health. 2015;12:701–7. 10.1123/jpah.2013-0212. 24906079 10.1123/jpah.2013-0212

