# Supplementary material for: Effects of a school-based cycling intervention on commuting to school behavior and device-measured activity in Spanish adolescents: the PACO cluster-randomized controlled trial
Source: Int J Behav Nutr Phys Act. 2026 Feb 24;23:29. doi: 10.1186/s12966-026-01893-1 (PMC13032254; doi:10.1186/s12966-026-01893-1)
Supplement: Supplementary file 1 — Supplementary Material 1. [file 12966_2026_1893_MOESM1_ESM.docx]

**Table S1.** CONSORT checklist.

|  | Section/topic | No | CONSORT 2025 checklist item description | Reported on page no. |
| --- | --- | --- | --- | --- |
|  | **Title and abstract** | | |  |
|  | Title and structured abstract | 1a | Identification as a randomised trial | 1 |
|  |  | 1b | Structured summary of the trial design, methods, results, and conclusions | 2 |
|  | **Open science** | | |  |
|  | Trial registration | 2 | Name of trial registry, identifying number (with URL) and date of registration | 1 |
|  | Protocol and statistical analysis plan | 3 | Where the trial protocol and statistical analysis plan can be accessed | 5 |
|  | Data sharing | 4 | Where and how the individual de-identified participant data (including data dictionary), statistical code and any other materials can be accessed | 23 |
|  | Funding and conflicts of interest | 5a | Sources of funding and other support (eg, supply of drugs), and role of funders in the design, conduct, analysis and reporting of the trial | 23 |
|  |  | 5b | Financial and other conflicts of interest of the manuscript authors | 23 |
|  | **Introduction** | | |  |
|  | Background and rationale | 6 | Scientific background and rationale | 4 |
|  | Objectives | 7 | Specific objectives related to benefits and harms | 4-5 |
|  | **Methods** | | |  |
|  | Patient and public involvement | 8 | Details of patient or public involvement in the design, conduct and reporting of the trial | 5 |
|  | Trial design | 9 | Description of trial design including type of trial (eg, parallel group, crossover), allocation ratio, and framework (eg, superiority, equivalence, non-inferiority, exploratory) | 5 |
|  | Changes to trial protocol | 10 | Important changes to the trial after it commenced including any outcomes or analyses that were not prespecified, with reason | 9 |
|  | Trial setting | 11 | Settings (eg, community, hospital) and locations (eg, countries, sites) where the trial was conducted | 5 |
|  | Eligibility criteria | 12a | Eligibility criteria for participants | 5 |
|  |  | 12b | If applicable, eligibility criteria for sites and for individuals delivering the interventions (eg, surgeons, physiotherapists) | NA |
|  | Intervention and comparator | 13 | Intervention and comparator with sufficient details to allow replication. If relevant, where additional materials describing the intervention and comparator (eg, intervention manual) can be accessed | 8-9 |
|  | Outcomes | 14 | Prespecified primary and secondary outcomes, including the specific measurement variable (eg, systolic blood pressure), analysis metric (eg, change from baseline, final value, time to event), method of aggregation (eg, median, proportion), and time point for each outcome | 7-8 |
|  | Harms | 15 | How harms were defined and assessed (eg, systematically, non-systematically) | NA |
|  | Sample size | 16a | How sample size was determined, including all assumptions supporting the sample size calculation | 10.3390/ijerph18042066 |
|  |  | 16b | Explanation of any interim analyses and stopping guidelines |  |
|  | Randomisation: |  |  | 5-6. 10.3390/ijerph18042066 |
|  | Sequence generation | 17a | Who generated the random allocation sequence and the method used |  |
|  |  | 17b | Type of randomisation and details of any restriction (eg, stratification, blocking and block size) |  |
|  |  |  |  |  |
|  | Allocation concealment mechanism | 18 | Mechanism used to implement the random allocation sequence (eg, central computer/telephone; sequentially numbered, opaque, sealed containers), describing any steps to conceal the sequence until interventions were assigned | 5-6 |
|  | Implementation | 19 | Whether the personnel who enrolled and those who assigned participants to the interventions had access to the random allocation sequence | 10.3390/ijerph18042066 |
|  | Blinding | 20a | Who was blinded after assignment to interventions (eg, participants, care providers, outcome assessors, data analysts) | NA |
|  |  | 20b | If blinded, how blinding was achieved and description of the similarity of interventions | NA |
|  | Statistical methods | 21a | Statistical methods used to compare groups for primary and secondary outcomes, including harms | 9-10 |
|  |  | 21b | Definition of who is included in each analysis (eg, all randomised participants), and in which group | 9-10 |
|  |  | 21c | How missing data were handled in the analysis | 9-10 |
|  |  | 21d | Methods for any additional analyses (eg, subgroup and sensitivity analyses), distinguishing prespecified from post hoc | 9-10 |
|  | **Results** | | |  |
|  | Participant flow, including flow diagram | 22a | For each group, the numbers of participants who were randomly assigned, received intended intervention, and were analysed for the primary outcome | 6 |
|  |  | 22b | For each group, losses and exclusions after randomisation, together with reasons | 5-6 |
|  | Recruitment | 23a | Dates defining the periods of recruitment and follow-up for outcomes of benefits and harms | 6 |
|  |  | 23b | If relevant, why the trial ended or was stopped | NA |
|  | Intervention and comparator delivery | 24a | Intervention and comparator as they were actually administered (eg, where appropriate, who delivered the intervention/comparator, how participants adhered, whether they were delivered as intended (fidelity)) | 8 |
|  |  | 24b | Concomitant care received during the trial for each group | 8 |
|  | Baseline data | 25 | A table showing baseline demographic and clinical characteristics for each group | 11-12 |
|  | Numbers analysed,  outcomes and estimation | 26 | For each primary and secondary outcome, by group:  ● the number of participants included in the analysis  ● the number of participants with available data at the outcome time point  ● result for each group, and the estimated effect size and its precision (such as 95% confidence interval)  ● for binary outcomes, presentation of both absolute and relative effect size | 6, 11-15 |
|  | Harms | 27 | All harms or unintended events in each group | NA |
|  | Ancillary analyses | 28 | Any other analyses performed, including subgroup and sensitivity analyses, distinguishing pre-specified from post hoc | 16 |
|  | **Discussion** | | |  |
|  | Interpretation | 29 | Interpretation consistent with results, balancing benefits and harms, and considering other relevant evidence | 17-20 |
|  | Limitations | 30 | Trial limitations, addressing sources of potential bias, imprecision, generalisability, and, if relevant, multiplicity of analyses | 20 |

Citation: Hopewell S, Chan AW, Collins GS, Hróbjartsson A, Moher D, Schulz KF, et al. CONSORT 2025 Statement: updated guideline for reporting randomised trials. BMJ. 2025; 388:e081123. <https://dx.doi.org/10.1136/bmj-2024-081123>
© 2025 Hopewell et al. This is an Open Access article distributed under the terms of the Creative Commons Attribution License (<https://creativecommons.org/licenses/by/4.0/>), which permits unrestricted use, distribution, and reproduction in any medium, provided the original work is properly cited.

*We strongly recommend reading this statement in conjunction with the CONSORT 2025 Explanation and Elaboration and/or the CONSORT 2025 Expanded Checklist for important clarifications on all the items. We also recommend reading relevant CONSORT extensions. See [www.consort-spirit.org](http://www.consort-spirit.org).

**Table S2.** TIDieR checklist for the PACO intervention.

| **Item number** | **Item** | **Where located **** | |
| --- | --- | --- | --- |
|  |  | Primary paper  (page or appendix  number) | Other ^†^ (details) |
|  | **BRIEF NAME** |  |  |
| **1.** | Provide the name or a phrase that describes the intervention. | 8 | Methods (intervention program) |
|  | **WHY** |  |  |
| **2.** | Describe any rationale, theory, or goal of the elements essential to the intervention. | 4 | Background |
|  | **WHAT** |  |  |
| **3.** | Materials: Describe any physical or informational materials used in the intervention, including those provided to participants or used in intervention delivery or in training of intervention providers. Provide information on where the materials can be accessed (e.g. online appendix, URL). | 8 | Methods (intervention program) |
| **4.** | Procedures: Describe each of the procedures, activities, and/or processes used in the intervention, including any enabling or support activities. | 8 | Methods (intervention program) |
|  | **WHO PROVIDED** |  |  |
| **5.** | For each category of intervention provider (e.g. psychologist, nursing assistant), describe their expertise, background and any specific training given. | 8 | Methods (intervention program) |
|  | **HOW** |  |  |
| **6.** | Describe the modes of delivery (e.g. face-to-face or by some other mechanism, such as internet or telephone) of the intervention and whether it was provided individually or in a group. | 5 & 8 | Methods (study design & intervention program) |
|  | **WHERE** |  |  |
| **7.** | Describe the type(s) of location(s) where the intervention occurred, including any necessary infrastructure or relevant features. | 5 & 8 | Methods (study design & intervention program) |
|  | **WHEN and HOW MUCH** |  |  |
| **8.** | Describe the number of times the intervention was delivered and over what period of time including the number of sessions, their schedule, and their duration, intensity or dose. | 8 | Methods (intervention program) |
|  | **TAILORING** |  |  |
| **9.** | If the intervention was planned to be personalised, titrated or adapted, then describe what, why, when, and how. | 8 | Methods (intervention program) & Chillón et al. (2021) |
|  | **MODIFICATIONS** |  |  |
| **10.^ǂ^** | If the intervention was modified during the course of the study, describe the changes (what, why, when, and how). | 7 & 8 | Methods (data collection & intervention program) |
|  | **HOW WELL** |  |  |
| **11.** | Planned: If intervention adherence or fidelity was assessed, describe how and by whom, and if any strategies were used to maintain or improve fidelity, describe them. | 9 | Methods (statistical analysis) |
| **12.^ǂ^** | Actual: If intervention adherence or fidelity was assessed, describe the extent to which the intervention was delivered as planned. | 16 & 19 | Results (per-protocol analysis) & Discussion (limitations) |

** **Authors** - use N/A if an item is not applicable for the intervention being described. **Reviewers** – use ‘?’ if information about the element is not reported/not sufficiently reported.

† If the information is not provided in the primary paper, give details of where this information is available. This may include locations such as a published protocol or other published papers (provide citation details) or a website (provide the URL).

ǂ If completing the TIDieR checklist for a protocol, these items are not relevant to the protocol and cannot be described until the study is complete.

* We strongly recommend using this checklist in conjunction with the TIDieR guide (see *BMJ* 2014;348:g1687) which contains an explanation and elaboration for each item.

* The focus of TIDieR is on reporting details of the intervention elements (and where relevant, comparison elements) of a study. Other elements and methodological features of studies are covered by other reporting statements and checklists and have not been duplicated as part of the TIDieR checklist. When a **randomised trial** is being reported, the TIDieR checklist should be used in conjunction with the CONSORT statement (see [www.consort-statement.org](http://www.consort-statement.org)) as an extension of **Item 5 of the CONSORT 2010 Statement.** When a **clinical trial** **protocol** is being reported, the TIDieR checklist should be used in conjunction with the SPIRIT statement as an extension of **Item 11 of the SPIRIT 2013 Statement** (see [www.spirit-statement.org](http://www.spirit-statement.org)). For alternate study designs, TIDieR can be used in conjunction with the appropriate checklist for that study design (see [www.equator-network.org](http://www.equator-network.org)).

**Table S3.** Moderation effect of gender and SES over the intervention effect on the usual mode of commuting to and from school.

|  | **Boys**  **OR (95% CI)** | **Girls**  **OR (95% CI)** |
| --- | --- | --- |
| Usual mode of commuting to school | 1.74  (0.48 to 6.29) | 0.54  (0.17 to 1.68) |
| Usual mode of commuting from school | 1.63  (0.45 to 5.85) | 0.74  (0.22 to 2.46) |
|  | **Medium**  **OR (95% CI)** | **High**  **OR (95% CI)** |
| Usual mode of commuting to school | 0.61  (0.18 to 2.14) | 1.24  (0.40 to 3.89) |
| Usual mode of commuting from school | 0.65  (0.18 to 2.29) | 1.57  (0.47 to 5.24) |

Note. OR and 95%CI are derived from logistic regression models including a three-way interaction between intervention group, time (baseline vs. follow-up), and gender or SES.

The outcome is the likelihood of engaging in active commuting (vs. passive commuting, reference category) to and from school.

Values above 1 indicate increased odds of active commuting in the given subgroup and time point compared to the reference condition (e.g., control group at follow-up). Models were adjusted for bike in good condition and home–school distance. In gender analyses, models were additionally adjusted for SES, and in SES analyses, models were additionally adjusted for gender.

**OR** = odds ratio; **CI** = confidence Interval; **SES** = socioeconomic status.

**
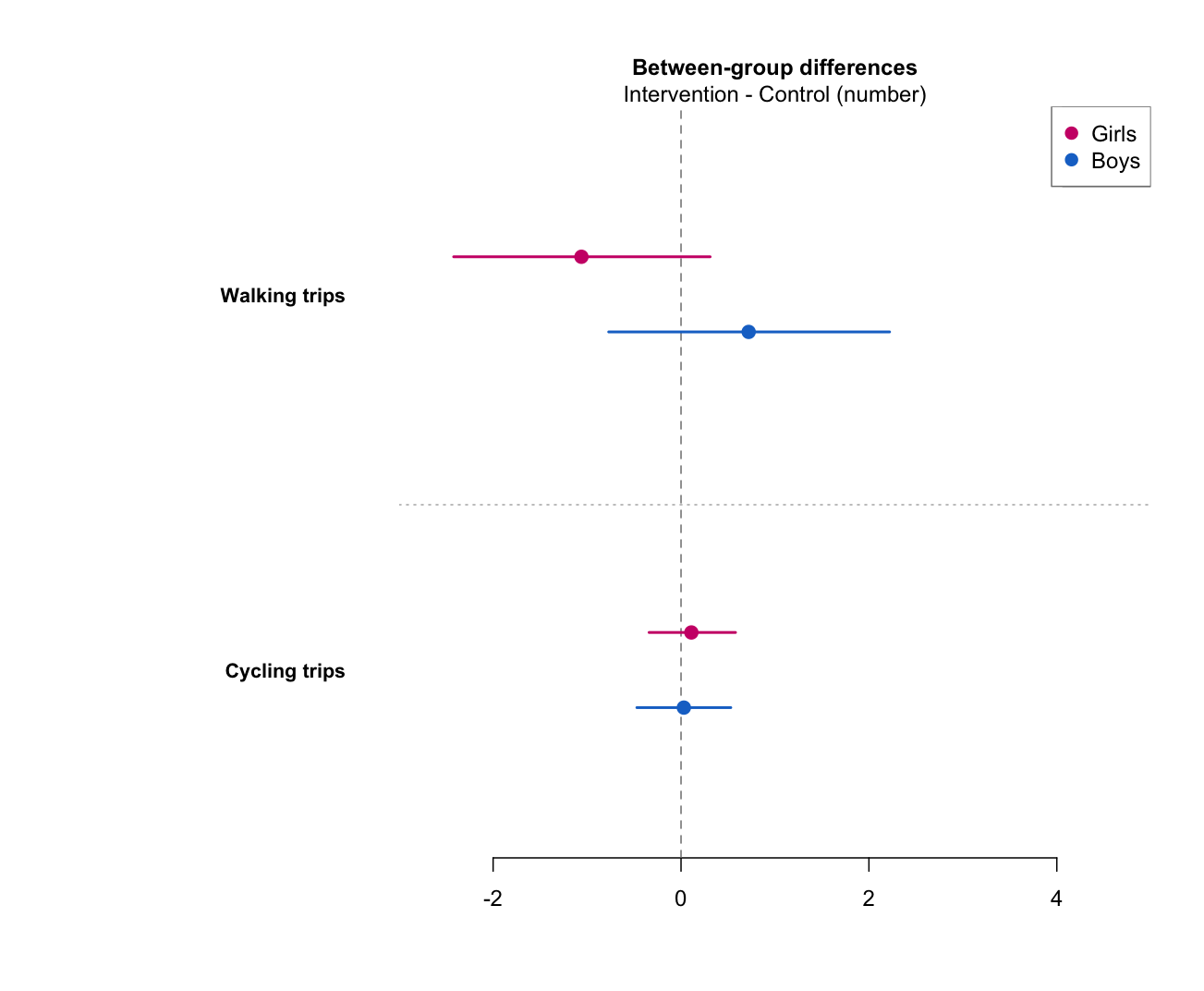
**

**Figure S1.** Moderation effect of gender over the intervention effect on the weekly frequency of ACS. Values represent mean differences with 95% confidence intervals for girls (pink) and boys (blue). Positive values indicate a higher number of trips in the intervention group compared with the control group.

Adjusted for: bike in good condition, SES, and home-school distance.

**
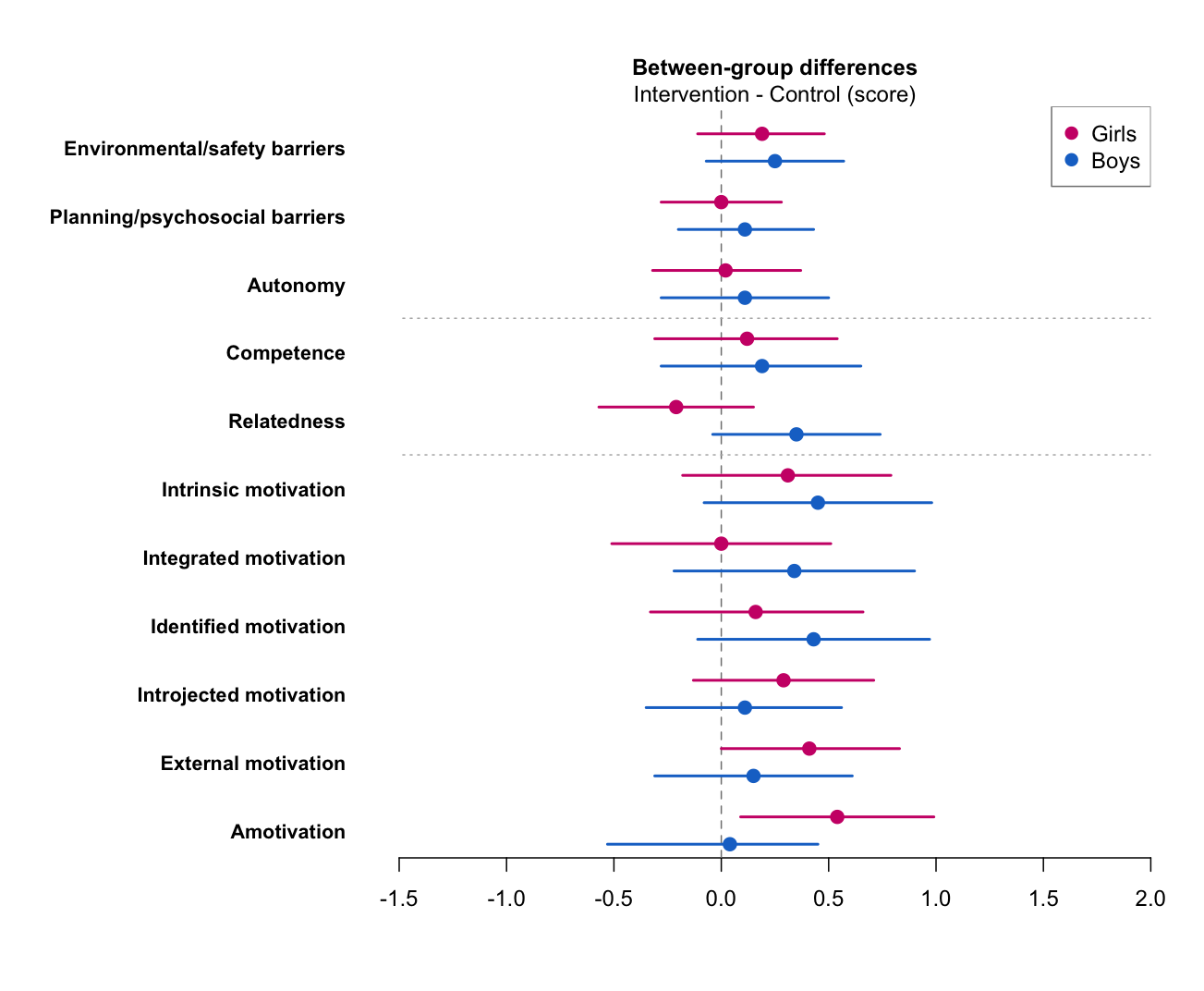
**

**Figure S2.** Moderation effect of gender over the intervention effect on ACS-related psychosocial outcomes. Values represent mean differences with 95% confidence intervals for girls (pink) and boys (blue). Positive values indicate a higher score in the intervention group compared with the control group.

Adjusted for: bike in good condition, SES, and home-school distance.


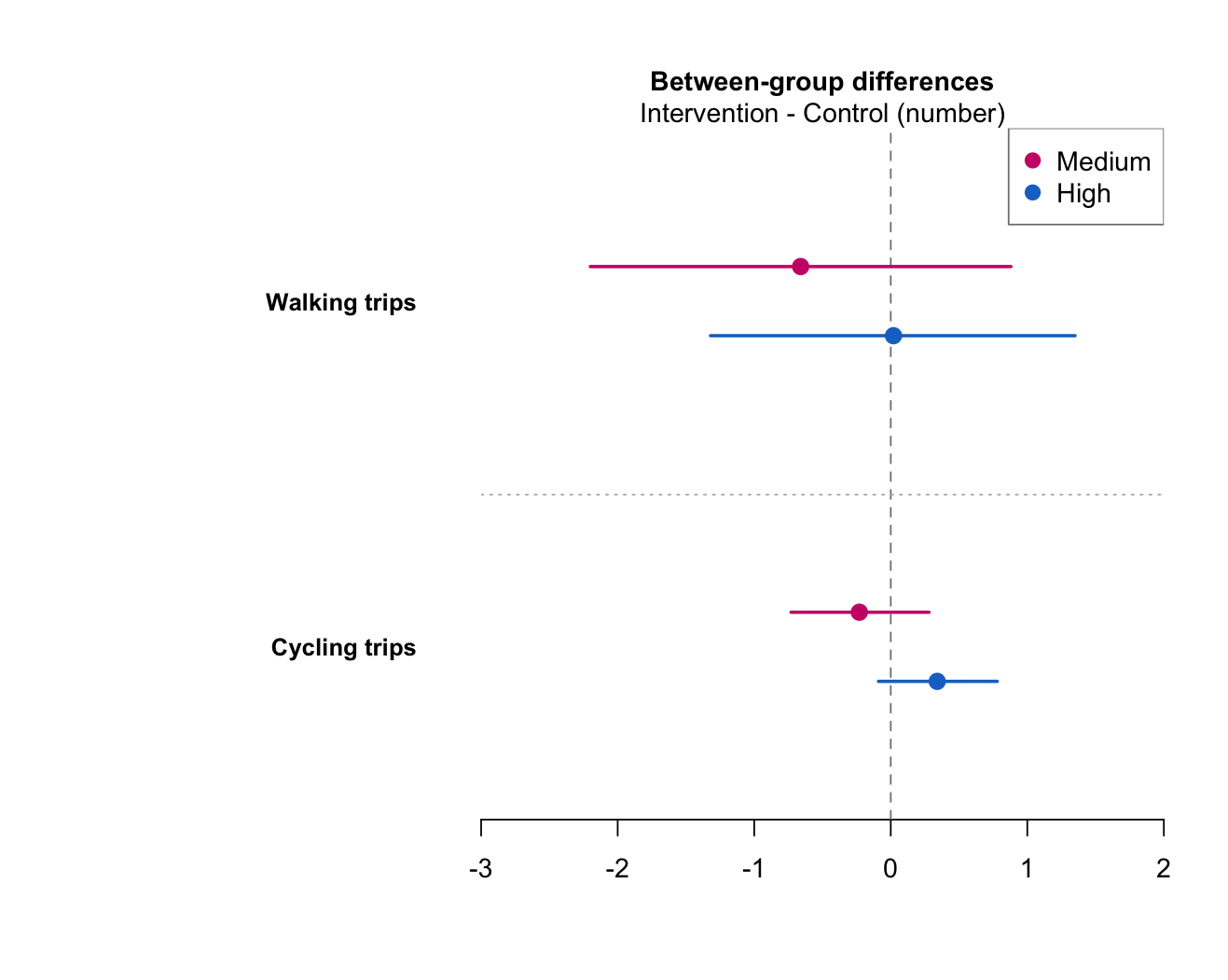


**Figure S3.** Moderation effect of SES over the intervention effect on the weekly frequency of ACS. Values represent mean differences with 95% confidence intervals for medium (pink) and high (blue). Positive values indicate a higher number of trips in the intervention group compared with the control group.

Adjusted for: gender, bike in good conditions, and home-school distance.


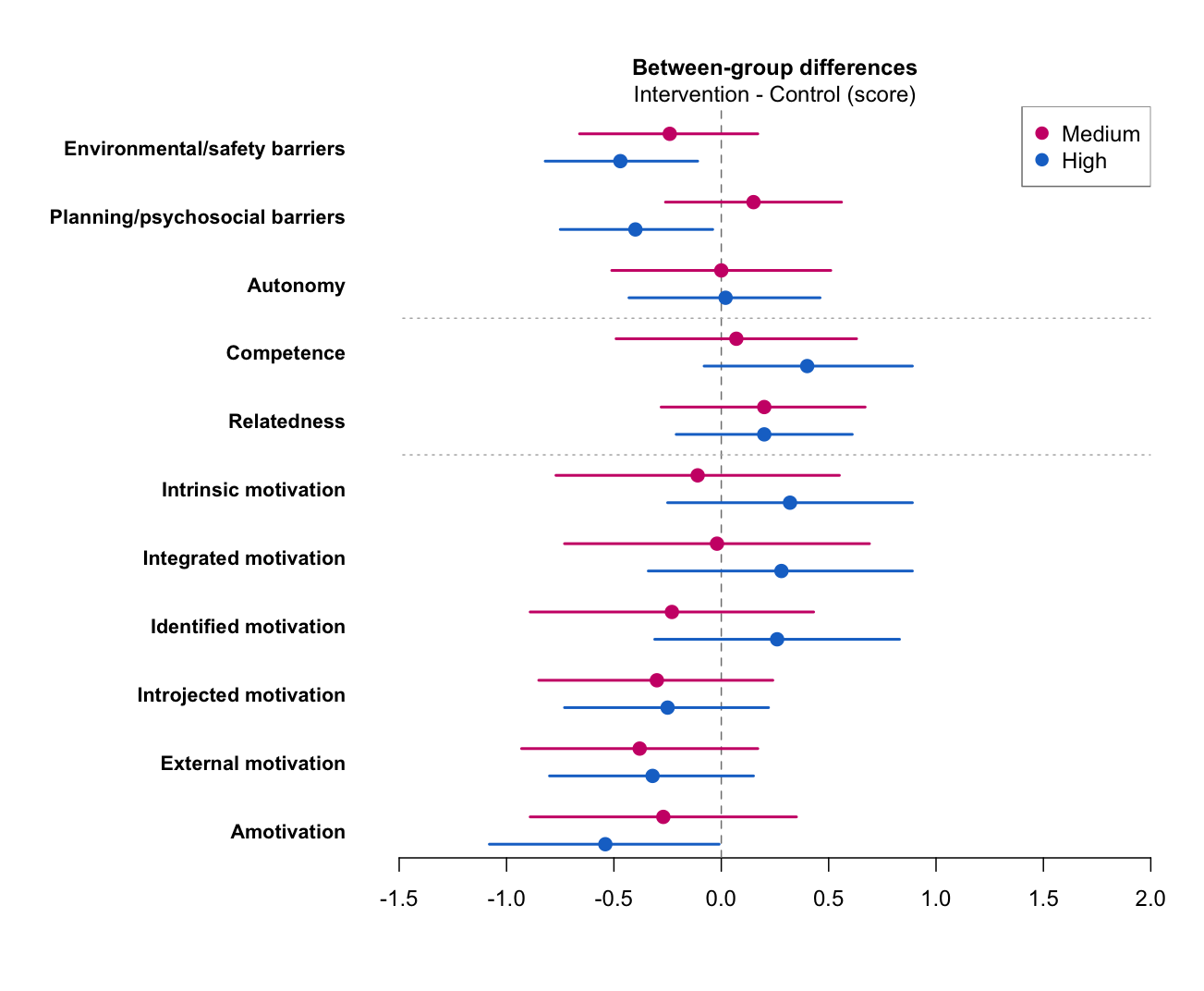


**Figure S4.** Moderation effect of SES over the intervention effect on ACS-related psychosocial outcomes. Values represent mean differences with 95% confidence intervals for medium (pink) and high (blue). Positive values indicate a higher score in the intervention group compared with the control group.

Adjusted for: gender, bike in good condition, and home-school distance.

**
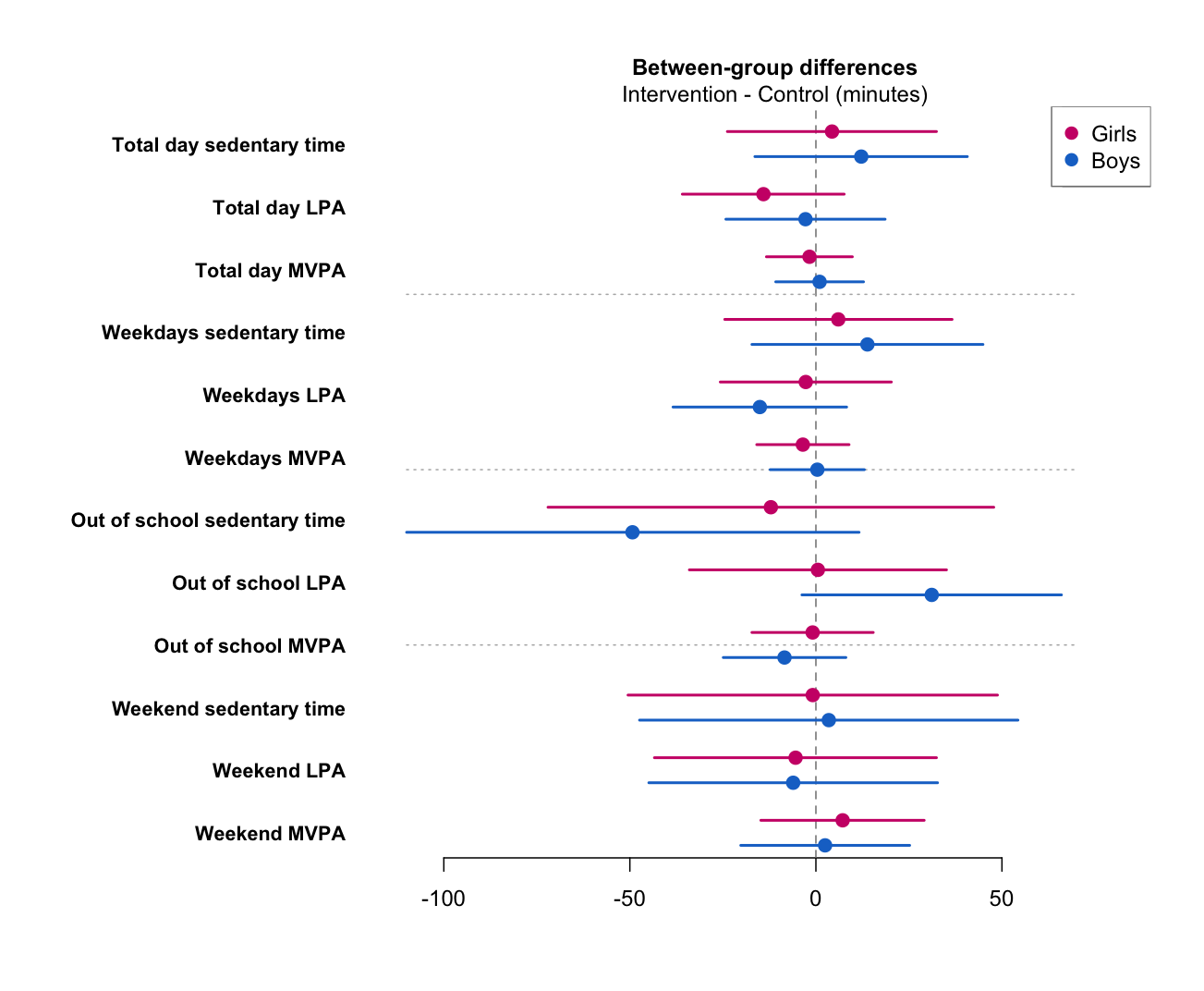
Figure S5.** Moderation effect of gender over the intervention effect on device-measured sedentary time, LPA, and MVPA during total day, weekdays, out of school, and weekend days. Values represent mean differences with 95% confidence intervals for girls (pink) and boys (blue). Positive values indicate higher minutes in the intervention group compared with the control group.

Adjusted for:bike in good condition, SES, and home-school distance.

**LPA** = light physical activity; **MVPA** = moderate-to-vigorous physical activity


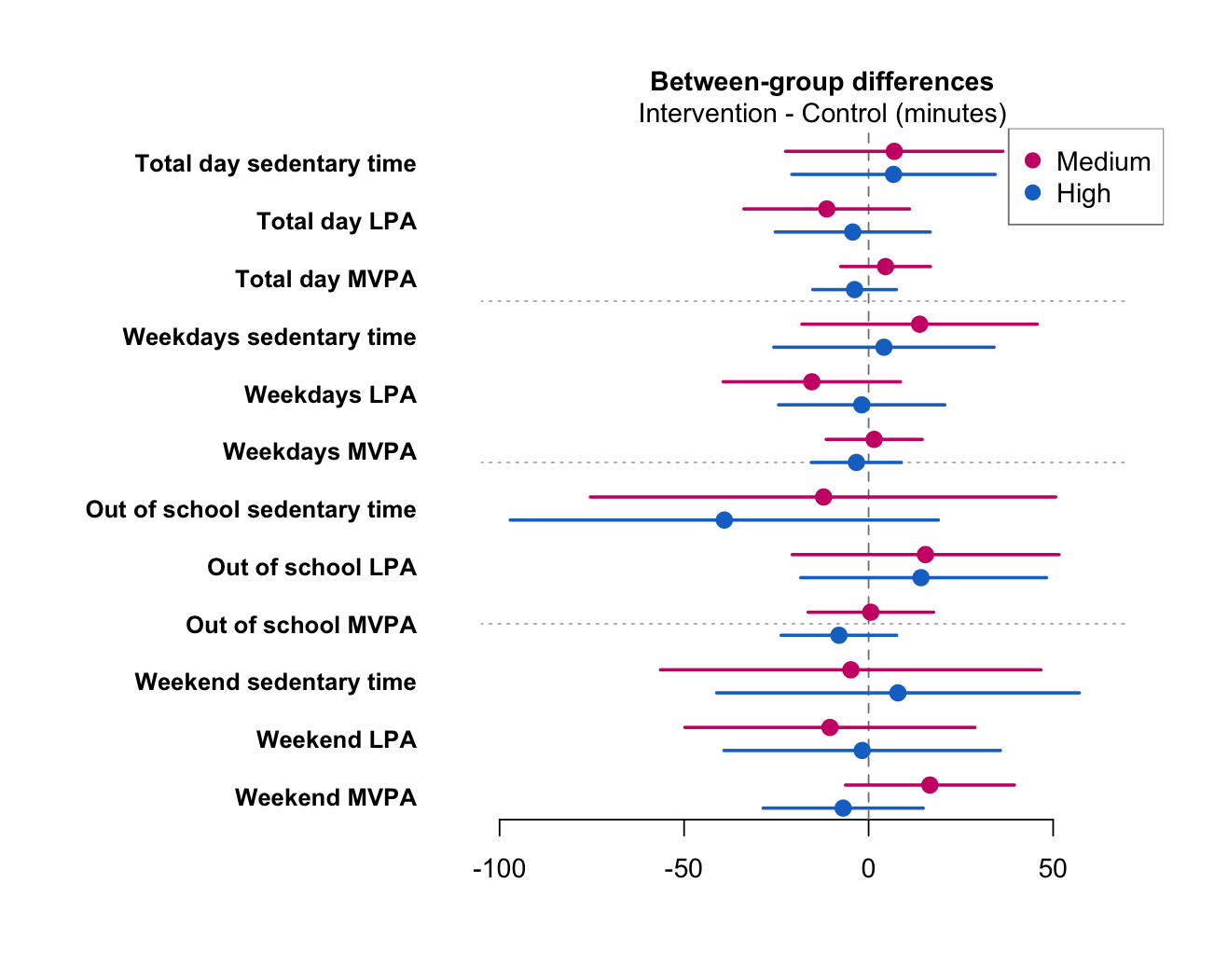


**Figure S6.** Moderation effect of gender over the intervention effect on device-measured sedentary time, LPA, and MVPA during total day, weekdays, out of school, and weekend days. Values represent mean differences with 95% confidence intervals for medium (pink) and high (blue). Positive values indicate higher minutes in the intervention group compared with the control group.

Adjusted for: gender, bike in good condition, and home-school distance.

**LPA** = light physical activity; **MVPA** = moderate-to-vigorous physical activity

**Table S4.** Descriptive characteristics of the participants included in the per-protocol analysis of the PACO study at baseline.

|  | **All**  **(n=131)** |  | **Intervention group**  **(n=52)** |  | **Control group**  **(n=79)** |  |
| --- | --- | --- | --- | --- | --- | --- |
| Age (years) | 14.4 ± 0.6 |  | 14.2 ± 0.5 |  | 14.4 ± 0.7 |  |
| Girls (%) | 80 (61.1) |  | 29 (55.8) |  | 51 (64.6) |  |
| **Usual mode of commuting** |  |  |  |  |  |  |
| Active commuting to school (%) | 81 (61.8) |  | 37 (71.2) |  | 44 (55.7) |  |
| Active commuting from school (%) | 87 (66.4) |  | 40 (76.9) |  | 47 (59.5) |  |
| **Weekly frequency of ACS** |  |  |  |  |  |  |
| Walking (nº trips) | 5.6 ± 4.3 |  | 6.4 ± 3.7 |  | 5.1 ± 4.6 |  |
| Cycling (nº trips) | 0.2 ± 1.0 |  | 0.0 ± 0.3 |  | 0.3 ± 1.3 |  |
| *Bike in good condition (%)* | | | | | | |
| Yes | 86 (65.6) |  | 30 (57.7) |  | 56 (70.9) |  |
| No | 45 (34.4) |  | 22 (42.3) |  | 23 (29.1) |  |
| *SES* |  |  |  |  |  |  |
| Medium (%) | 58 (44.3) |  | 20 (38.5) |  | 38 (48.1) |  |
| High (%) | 73 (55.7) |  | 32 (61.5) |  | 41 (49.9) |  |
| Home-school distance (km) | 3.7 ± 8.2 |  | 3.8 ± 1.2 |  | 3.7 ± 4.7 |  |
| **Perceived barriers to ACS** | | | | | | |
| Environmental/safety barriers (score) | 1.8 ± 0.7 |  | 1.7 ± 0.6 |  | 1.9 ± 0.8 |  |
| Planning/psychosocial barriers (score) | 1.9 ± 0.7 |  | 1.8 ± 0.6 |  | 1.9 ± 0.7 |  |
| **Basic psychological needs related to ACS** | | | | | | |
| Autonomy (score) | 3.7 ± 1.2 |  | 4.1 ± 0.9 |  | 3.6 ± 1.2 |  |
| Competence (score) | 4.0 ± 1.3 |  | 4.4 ± 0.9 |  | 3.7 ± 1.5 |  |
| Relatedness (score) | 4.4 ± 0.9 |  | 4.5 ± 0.7 |  | 4.3 ± 1.0 |  |
| **Motivation for ACS** | | | | | | |
| Intrinsic (score) | 2.3 ± 1.4 |  | 2.6 ± 1.3 |  | 2.2 ± 1.4 |  |
| Integrated (score) | 1.9 ± 1.4 |  | 2.1 ± 1.3 |  | 1.7 ± 1.4 |  |
| Identified (score) | 2.1 ± 1.3 |  | 2.2 ± 1.1 |  | 2.0 ± 1.4 |  |
| Introjected (score) | 0.4 ± 0.6 |  | 0.4 ± 0.6 |  | 0.5 ± 0.6 |  |
| External (score) | 0.6 ± 0.8 |  | 0.5 ± 0.7 |  | 0.6 ± 0.8 |  |
| Amotivation (score) | 0.7 ± 0.9 |  | 0.6 ± 0.8 |  | 0.7 ± 0.9 |  |

Results are presented as mean ± SD for continuous variables and as n (%) for categorical outcomes.

Basic psychological needs related to ACS ranges from 0 (lower basic psychological needs related to ACS) to 5 (higher basic psychological needs related to ACS); perceived barriers to ACS ranges from1 (lower perception of barriers to ACS) to 4 (higher perception of barriers to ACS; motivation for ACS ranges from 0 (lower motivation for ACS) to 4 (higher motivation for ACS), it is important to note that amotivation’s interpretation is inversely.

**n** = sample size; **%** = percentage; **SD** = Standard deviation; **SES** = socioeconomic status; **km** = kilometer; **nº** = number; **ACS** = active commuting to and from school.

**Table S5.** Descriptive characteristics of the participants included in the per-protocol analysis of the PACO study at baseline with valid accelerometry data.

|  | **All**  **(n=61)** |  | **Intervention group**  **(n=25)** |  | **Control group**  **(n=36)** |  |
| --- | --- | --- | --- | --- | --- | --- |
| Age (years) | 14.3 ± 0.5 |  | 14.3 ± 0.5 |  | 14.4 ± 0.5 |  |
| Girls (%) | 34 (55.7) |  | 13 (52.0) |  | 21 (58.3) |  |
| *Bike in good conditions* | | | | | | |
| Yes | 41 (67.2) |  | 17 (68.0) |  | 24 (66.7) |  |
| No | 20 (32.8) |  | 8 (32.0) |  | 12 (33.3) |  |
| *SES* |  |  |  |  |  |  |
| Medium (%) | 28 (45.9) |  | 9 (36.0) |  | 19 (52.8) |  |
| High (%) | 33 (44.1) |  | 16 (64.0) |  | 17 (47.2) |  |
| Home-school distance (km) | 4.7 ± 11.2 |  | 6.0 ± 16.8 |  | 3.8 ± 4.6 |  |
| **Total day** | | | | | | |
| Wear time (min) | 801.5 ± 99.1 |  | 766.2 ± 103.1 |  | 825.9 ± 89.8 |  |
| Sedentary time (min) | 613.7 ± 90.9 |  | 581.8 ± 91.9 |  | 635.9 ± 84.7 |  |
| LPA (min) | 151.9 ± 43.8 |  | 151.6 ± 44.3 |  | 152.3 ± 44.1 |  |
| MVPA (min) | 35.7 ± 18.4 |  | 32.8 ± 17.0 |  | 37.8 ± 19.2 |  |
| **Weekdays** | | | | | | |
| Wear time (min) | 828.8 ± 124.8 |  | 785.5 ± 120.6 |  | 858.9 ± 120.2 |  |
| Sedentary time (min) | 636.7 ± 113.2 |  | 594.2 ± 115.3 |  | 666.2 ± 103.2 |  |
| LPA (min) | 152.2 ± 45.9 |  | 152.2 ± 24.5 |  | 154.2 ± 47.0 |  |
| MVPA (min) | 37.9 ± 20.6 |  | 37.0 ± 21.2 |  | 38.5 ± 20.6 |  |
| **Out of school** | | | | | | |
| Wear time (min) |  |  | 530.9 ± 214.0 |  | 583.5 ± 222.7 |  |
| Sedentary time (min) | 385.9 ± 105.3 |  | 346.7 ± 92.2 |  | 413.2 ± 106.9 |  |
| LPA (min) | 141.7 ± 43.9 |  | 152.6 ± 39.8 |  | 134.1 ± 45.5 |  |
| MVPA (min) | 33.9 ± 18.6 |  | 30.7 ± 17.8 |  | 36.2 ± 19.0 |  |
| **Weekends** |  |  |  |  |  |  |
| Wear time (min) | 733.2 ± 120.4 |  | 717.9 ± 126.8 |  | 743.7 ± 116.4 |  |
| Sedentary time (min) | 556.3 ± 115.9 |  | 550.9 ± 94.8 |  | 560.1 ± 129.9 |  |
| LPA (min) | 146.4 ± 57.4 |  | 144.9 ± 62.7 |  | 147.5 ± 54.4 |  |
| MVPA (min) | 30.4 ± 28.2 |  | 22.1 ± 19.7 |  | 36.2 ± 31.9 |  |

Results are presented as mean ± SD for continuous variables and as n (%) for categorical outcomes.

Basic psychological needs related to ACS ranges from 0 (lower basic psychological needs related to ACS) to 5 (higher basic psychological needs related to ACS); perceived barriers to ACS ranges from1 (lower perception of barriers to ACS) to 4 (higher perception of barriers to ACS; motivation for ACS ranges from 0 (lower motivation for ACS) to 4 (higher motivation for ACS), it is important to note that amotivation’s interpretation is inversely.

**n =** sample size**; %=** percentage; **SD** = Standard deviation; **SES** = socioeconomic status; **km** = kilometer; **nº** = number; **LPA** = light physical activity; **MVPA** = moderate-to-vigorous physical activity; **min** = minutes.


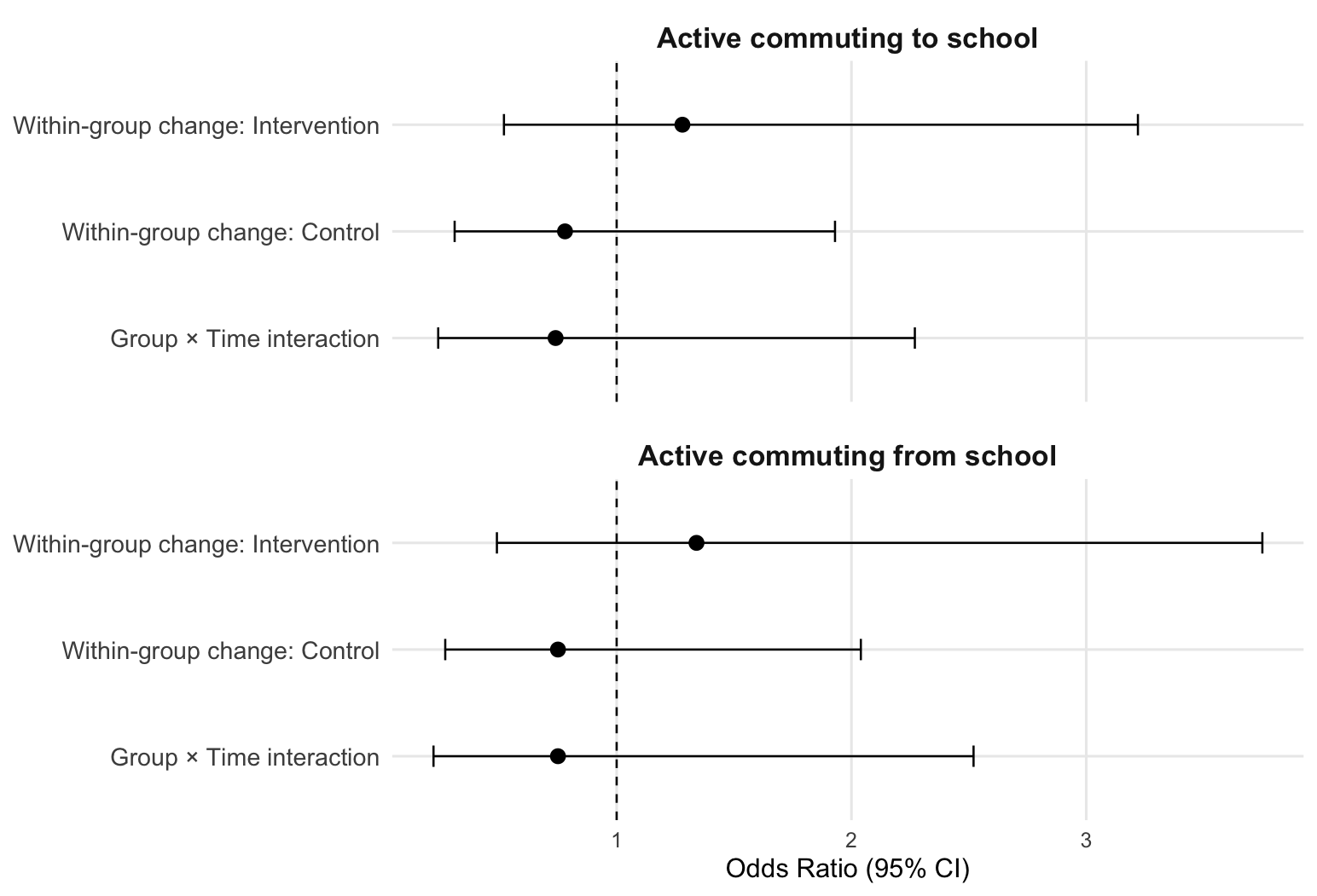


**Figure S7**. Per-protocol within- and between-group changes in the usual mode of commuting to and from school from baseline to follow-up.

Odds ratios (OR) and 95% confidence intervals for between-group, within-group, and interaction effects. Reference: passive commuting.

Adjusted for: gender, bike in good condition, SES, and home-school distance.

**Table S6.** Per-protocol effects of the intervention on weekly frequency of ACS, basic psychological needs related to ACS, perceived barriers to ACS, and motivational outcomes for ACS from baseline to follow-up.

| **Outcome** | **Intervention group**  **Mean (95% CI)**  **(n=52)**  (**Follow-up** *minus* **baseline**) | **Control group**  **Mean (95% CI)**  **(n=79)**  (**Follow-up** *minus* **baseline**) | **Difference in change from baseline to follow-up**  **Mean (95% CI)**  (**Intervention** *minus* **control**) | **Group X time**  *p*-**value** |
| --- | --- | --- | --- | --- |
| **Weekly frequency of ACS** | | | | |
| *Walking trips* | | | | |
| Number of trips | 0.35 (-0.52 to 1.22) | 0.18 (-0.54 to 0.89) | 0.17 (-0.95 to 1.31) | 0.762 |
| Cohen’s d | 0.05 (-0.34 to 0.44) | 0.02 (-0.29 to 0.33) | 0.05 (-0.29 to 0.40) |  |
| *Cycling trips* | | | | |
| Number of trips | -0.03 (-0.26 to 0.19) | 0.06 (-0.12 to 0.25) | -0.10 (-0.40 to 0.19) | 0.496 |
| Cohen’s d | -0.03 (-0.42 to 0.36) | 0.04 (-0.28 to 0.35) | -0.11 (-0.45 to 0.24) |  |
| **Perceived barriers to ACS** | | | | |
| *Environmental/safety barriers* | | | | |
| Score | **0.27 (0.08 to 0.48)** | -0.11 (-0.28 to 0.06) | **0.39 (0.12 to 0.65)** | 0.005 |
| Cohen’s d | **0.39 (0.00 to 0.78)** | -0.12 (-0.44 to 0.19) | **0.49 (0.14 to 0.89)** |  |
| *Planning/psychosocial barriers* | | | | |
| Score | 0.20 (-0.01 to 0.41) | 0.02 (-0.15 to 0.19) | 0.18 (-0.09 to 0.46) | 0.184 |
| Cohen’s d | 0.28 (-0.08 to 0.67) | 0.02 (-0.29 to 0.34) | 0.25 (-0.10 to 0.59) |  |
| **Basic psychological needs related to ACS** | | | | |
| *Autonomy* | | | | |
| Score | 0.12 (-0.13 to 0.38) | 0.14 (-0.07 to 0.35) | -0.02 (-0.35 to 0.31) | 0.168 |
| Cohen’s d | 0.11 (-0.28 to 0.49) | 0.09 (-0.22 to 0.41) | -0.02 (-0.37 to 0.32) |  |
| *Competence* | | | | |
| Score | -0.13 (-0.41 to 0.15) | 0.13 (-0.10 to 0.36) | -0.26 (-0.63 to 0.10) | 0.158 |
| Cohen’s d | -0.06 (-0.45 to 0.32) | 0.06 (-0.26 to 0.37) | -0.27 (-0.62 to 0.08) |  |
| *Relatedness* |  |  |  |  |
| Score | -0.22 (-0.45 to 0.02) | 0.00 (-0.19 to 0.19) | -0.21 (-0.52 to 0.09) | 0.171 |
| Cohen’s d | -0.22 (-0.61 to 0.17) | 0.00 (-0.31 to 0.31) | -0.24 (-0.59 to 0.11) |  |
| **Motivation for ACS** | | | | |
| *Intrinsic motivation* | | | | |
| Score | 0.09 (-0.24 to 0.43) | 0.24 (-0.04 to 0.51) | -0.14 (-0.57 to 0.29) | 0.516 |
| Cohen’s d | 0.06 (-0.33 to 0.44) | 0.21 (-0.11 to 0.52) | -0.12 (-0.47 to 0.22) |  |
| *Integrated motivation* | | | | |
| Score | 0.15 (-0.21 to 0.51) | **0.32 (0.02 to 0.61)** | -0.16 (-0.63 to 0.30) | 0.482 |
| Cohen’s d | 0.07 (-0.32 to 0.46) | 0.12 (-0.19 to 0.42) | -0.13 (-0.47 to 0.22) |  |
| *Identified motivation* | | | | |
| Score | 0.20 (-0.14 to 0.53) | 0.22 (-0.05 to 0.49) | -0.02 (-0.45 to 0.41) | 0.915 |
| Cohen’s d | 0.20 (-0.19 to 0.58) | 0.12 (-0.19 to 0.44) | -0.02 (-0.36 to 0.33) |  |
| *Introjected motivation* | | | | |
| Score | **0.53 (0.25 to 0.80)** | **0.24 (0.02 to 0.47)** | 0.29 (-0.07 to 0.74) | 0.115 |
| Cohen’s d | **0.57 (0.17 to 0.96)** | 0.21 (-0.11 to 0.52) | 0.28 (-0.06 to 0.63) |  |
| *External motivation* | | | | |
| Score | **0.47 (0.19 to 0.75)** | 0.09 (-0.14 to 0.32) | **0.38 (0.02 to 0.74)** | 0.037 |
| Cohen’s d | 0.38 (-0.01 to 0.78) | 0.08 (-0.23 to 0.40) | **0.38 (0.03 to 0.73)** |  |
| *Amotivation* | | | | |
| Score | **0.39 (0.08 to 0.71)** | -0.05 (-0.31 to 0.20) | **0.45 (0.04 to 0.85)** | 0.030 |
| Cohen’s d | 0.39 (-0.01 to 0.78) | -0.04 (-0.36 to 0.27) | **0.39 (0.04 to 0.74)** |  |

**ACS** = active commuting to and from school; **CI** = confidence interval; **n** = sample size

Notes. All data presented were adjusted for gender, bike in good condition, SES, and home-school distance. Bold values indicate significance (p < 0.05).

**Table S7.** Per-protocol effects of the intervention on daily device-measured sedentary time and physical activity, during weekdays, out of school, and during weekends from baseline to follow-up.

| **Outcome** | **Intervention group**  **Mean (95% CI)**  **(n=25)**  (**Follow-up** *minus* **baseline**) | **Control group**  **Mean (95% CI)**  **(n=36)**  (**Follow-up** *minus* **baseline**) | **Difference in change from baseline to follow-up**  **Mean (95% CI)**  (**Intervention** *minus* **control**) | **Group X time**  *p*-**value** |
| --- | --- | --- | --- | --- |
| **Total day** | | | | |
| Sedentary time (min) | -11.42 (-31.95 to 9.11) | -5.51 (-23.17 to 12.12) | -5.91 (-33.00 to 21.28) | 0.664 |
| Cohen’s d | -0.19 (-0.76 to 0.38) | -0.14 (-0.61 to 0.33) | -0.11 (-0.62 to 0.40) |  |
| LPA (min) | 8.35 ( -7.32 to 24.07) | 3.77 (-9.67 to 17.22) | 4.57 (-16.14 to 25.23) | 0.659 |
| Cohen’s d | 0.14 (-0.43 to 0.70) | 0.06 (-0.41 to 0.53) | 0.14 (-0.37 to 0.65) |  |
| MVPA (min) | 2.89 (-7.08 to 12.85) | -0.10 (-8.60 to 8.41) | 2.98 (-10.16 to 16.19) | 0.651 |
| Cohen’s d | 0.12 (-0.45 to 0.68) | 0.00 (-0.47 to 0.47) | 0.12 (-0.39 to 0.63) |  |
| **Weekdays** | | | | |
| Sedentary time (min) | -13.87 (-38.33 to 10.61) | -9.72 (-30.66 to 11.27) | -4.15 (-36.34 to 28.00) | 0.797 |
| Cohen’s d | -0.20 (-0.76 to 0.37) | -0.11 (-0.58 to 0.36) | -0.06 (-0.58 to 0.45) |  |
| LPA (min) | 11.06 (-5.23 to 27.49) | 8.46 (-5.51 to 22.45) | 2.60 (-18.91 to 24.13) | 0.809 |
| Cohen’s d | 0.20 (-0.36 to 0.77) | 0.13 (-0.34 to 0.60) | 0.06 (-0.45 to 0.57) |  |
| MVPA (min) | 2.82 (-8.82 to 14.58) | 1.25 (-8.66 to 11.25) | 1.57 (-13.77 to 16.93) | 0.838 |
| Cohen’s d | 0.09 (-0.48 to 0.66) | 0.04 (-0.43 to 0.51) | 0.05 (-0.46 to 0.56) |  |
| **Out of school** | | | | |
| Sedentary time (min) | 20.44 (-38.63 to 79.42) | 18.93 (-27.80 to 65.66) | 1.44 (-71.52 to 74.48) | 0.969 |
| Cohen’s d | 0.13 (-0.43 to 0.70) | 0.12 (-0.35 to 0.59) | 0.01 (-0.50 to 0.52) |  |
| LPA (min) | **64.92 (38.33 to 91.67)** | **64.93 (44.21 to 85.72)** | -0.01 (-32.36 to 32.36) | 0.999 |
| Cohen’s d | **0.61 (0.03 to 1.19)** | **0.58 (0.10 to 1.06)** | 0.00 (-0.51 to 0.51) |  |
| MVPA (min) | 11.89 (-4.09 to 27.91) | -1.29 (-13.95 to 11.40) | 13.24 (-6.61 to 33.00) | 0.188 |
| Cohen’s d | 0.29 (-0.28 to 0.86) | -0.03 (-0.50 to 0.44) | 0.34 (-0.17 to 0.85) |  |
| **Weekend** | | | | |
| Sedentary time (min) | -5.31 (-39.68 to 29.04) | 7.21 (-21.80 to 36.22) | -12.5 (-57.55 to 32.43) | 0.579 |
| Cohen’s d | -0.05 (-0.62 to 0.52) | 0.06 (-0.41 to 0.53) | -0.15 (-0.66 to 0.36) |  |
| LPA (min) | 2.19 (-24.11 to 28.49) | -4.42 (-26.61 to 17.85) | 6.60 (-27.84 to 41.07) | 0.702 |
| Cohen’s d | 0.02 (-0.54 to 0.59) | -0.05 (-0.52 to 0.41) | 0.10 (-0.41 to 0.61) |  |
| MVPA (min) | 3.14 (-11.72 to 18.02) | -2.58 (-15.10 to 9.97) | 5.72 (-13.77 to 25.28) | 0.558 |
| Cohen’s d | 0.09 (-0.47 to 0.66) | -0.07 (-0.54 to 0.40) | 0.15 (-0.36 to 0.66) |  |

**CI** = confidence interval; **LPA** = light physical activity; **MVPA** = moderate-to-vigorous physical activity; **min** = minutes; **n** = sample size

Notes. All data presented were adjusted for gender, bike in good condition, SES, home-school distance, and wear time in each examined context (i.e., total wear time, weekdays wear time, out of school wear time, and weekends wear time). Bold values indicate significance (p < 0.05).

**Table S8.** Per-protocol moderation effect of gender and SES over the intervention effect on the usual mode of commuting to and from school.

|  | **Boys**  **OR (95% CI)** | **Girls**  **OR (95% CI)** |
| --- | --- | --- |
| Usual mode of commuting to school | 1.00  (0.16 to 6.00) | 0.63  (0.14 to 2.54) |
| Usual mode of commuting from school | 0.77  (0.13 to 4.59) | 0.73  (0.14 to 3.91) |
|  | **Medium**  **OR (95% CI)** | **High**  **OR (95% CI)** |
| Usual mode of commuting to school | 0.62  (0.13 to 3.09) | 0.86  (0.48 to 4.17) |
| Usual mode of commuting from school | 0.69  (0.14 to 3.47) | 0.86  (0.12 to 6.34) |

Note. OR and 95%CI are derived from logistic regression models including a three-way interaction between intervention group, time (baseline vs. follow-up), and gender or FAS.

The outcome is the likelihood of engaging in active commuting (vs. passive commuting, reference category) to and from school.

Values above 1 indicate increased odds of active commuting in the given subgroup and time point compared to the reference condition (e.g., control group at follow-up). Models were adjusted for bike in good condition and home–school distance. In gender analyses, models were additionally adjusted for SES, and in SES analyses, models were additionally adjusted for gender.

**OR** = odds ratio; **CI** = confidence Interval; **SES** = socioeconomic status.


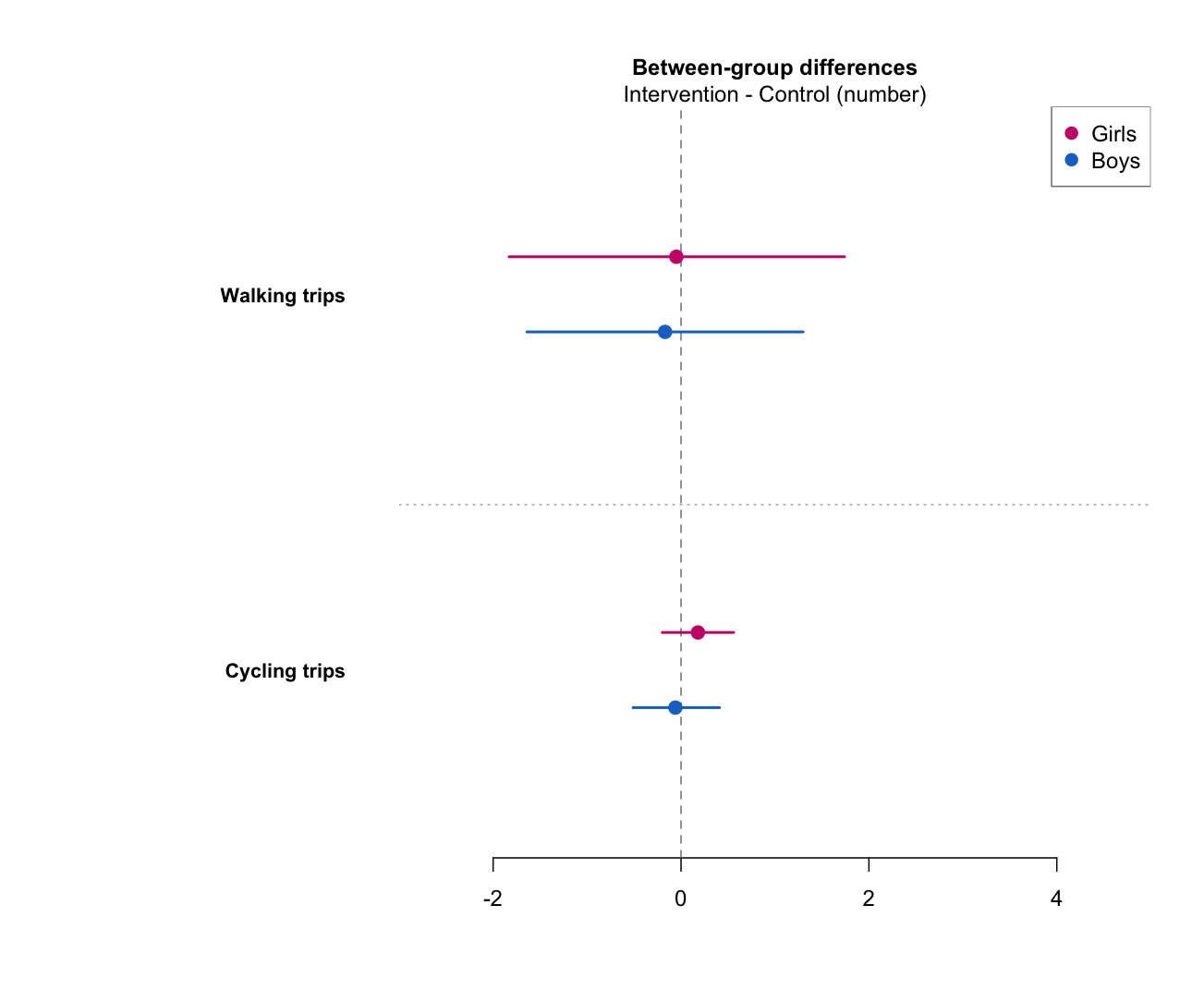


**Figure S8.** Per-protocol moderation effect of gender over the intervention effect on the weekly frequency of ACS. Values represent mean differences with 95% confidence intervals for girls (pink) and boys (blue). Positive values indicate a higher number of trips in the intervention group compared with the control group.

Adjusted for:bike in good condition, SES, and home-school distance.


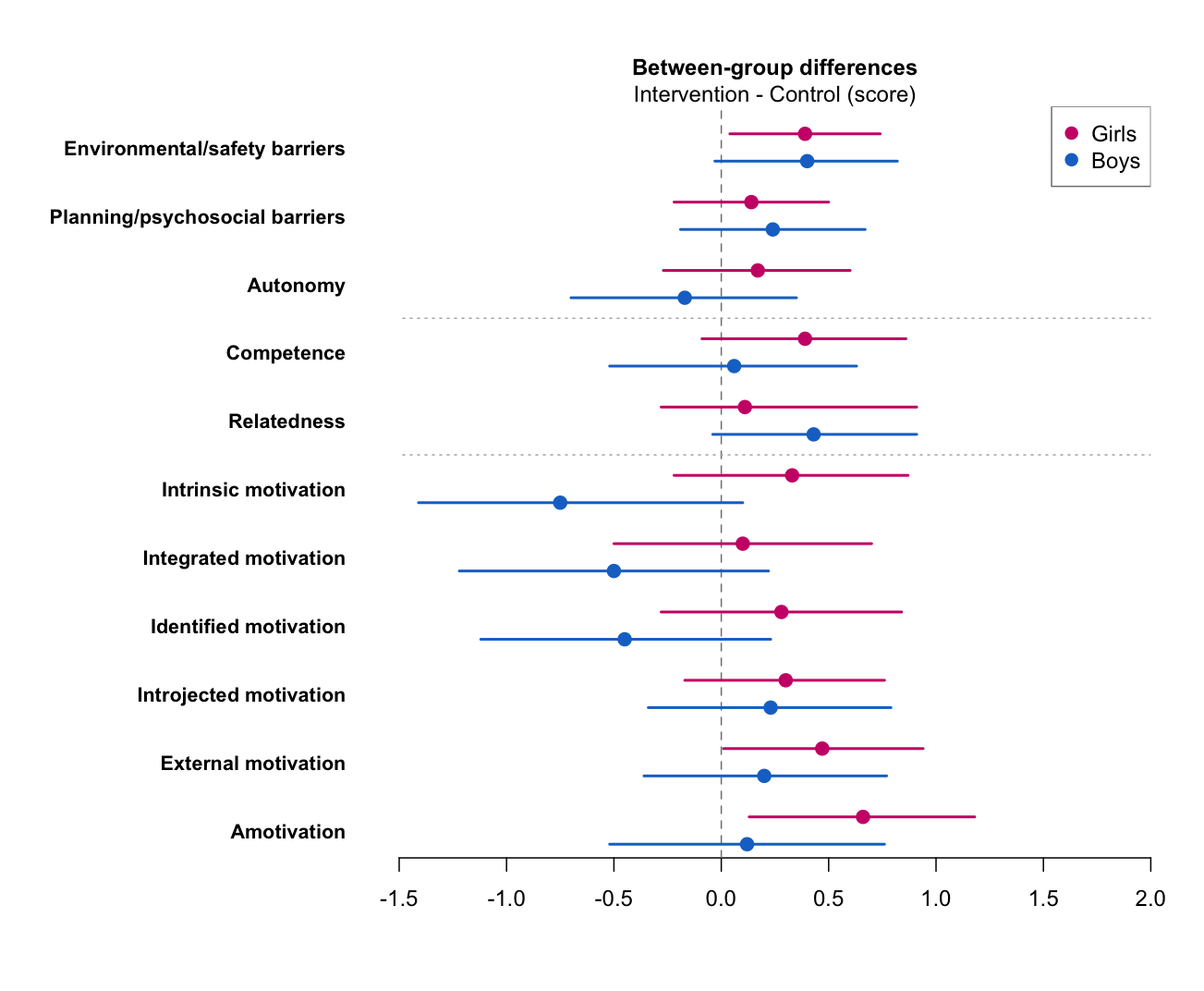


**Figure S9.** Per-protocol moderation effect of gender over the intervention effect on ACS-related psychosocial outcomes. Values represent mean differences with 95% confidence intervals for girls (pink) and boys (blue). Positive values indicate a higher score in the intervention group compared with the control group.

Adjusted for: bike in good condition, SES, and home-school distance.


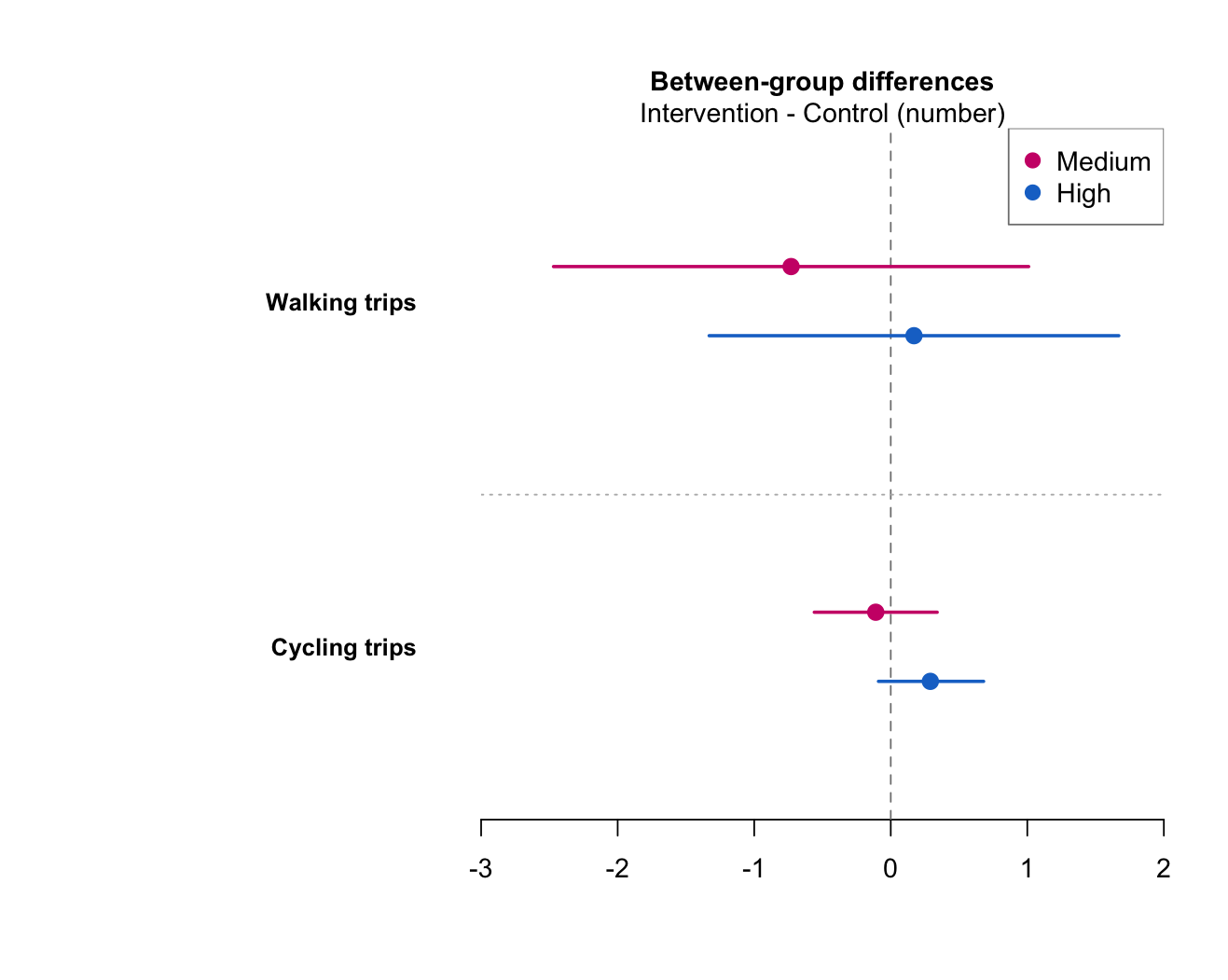


**Figure S10.** Per-protocol moderation effect of SES over the intervention effect on the weekly frequency of ACS. Values represent mean differences with 95% confidence intervals for medium (pink) and high (blue). Positive values indicate a higher number of trips in the intervention group compared with the control group.

Adjusted for: gender, bike in good condition, and home-school distance.

**
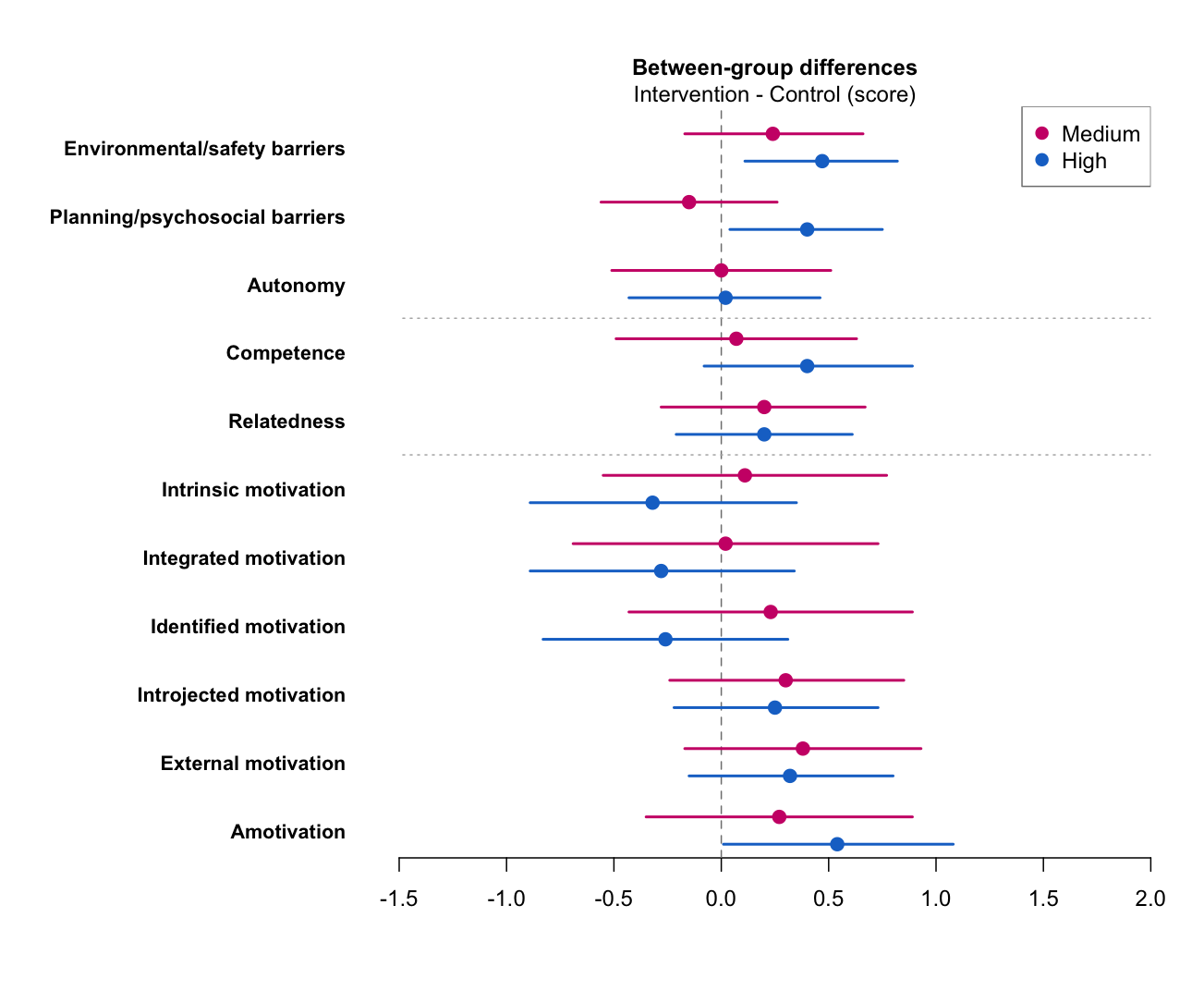
Figure S11.** Per-protocol moderation effect of SES over the intervention effect on ACS-related psychosocial outcomes. Values represent mean differences with 95% confidence intervals for medium (pink) and high (blue). Positive values indicate a higher score in the intervention group compared with the control group.

Adjusted for: gender, bike in good condition, and home-school distance.


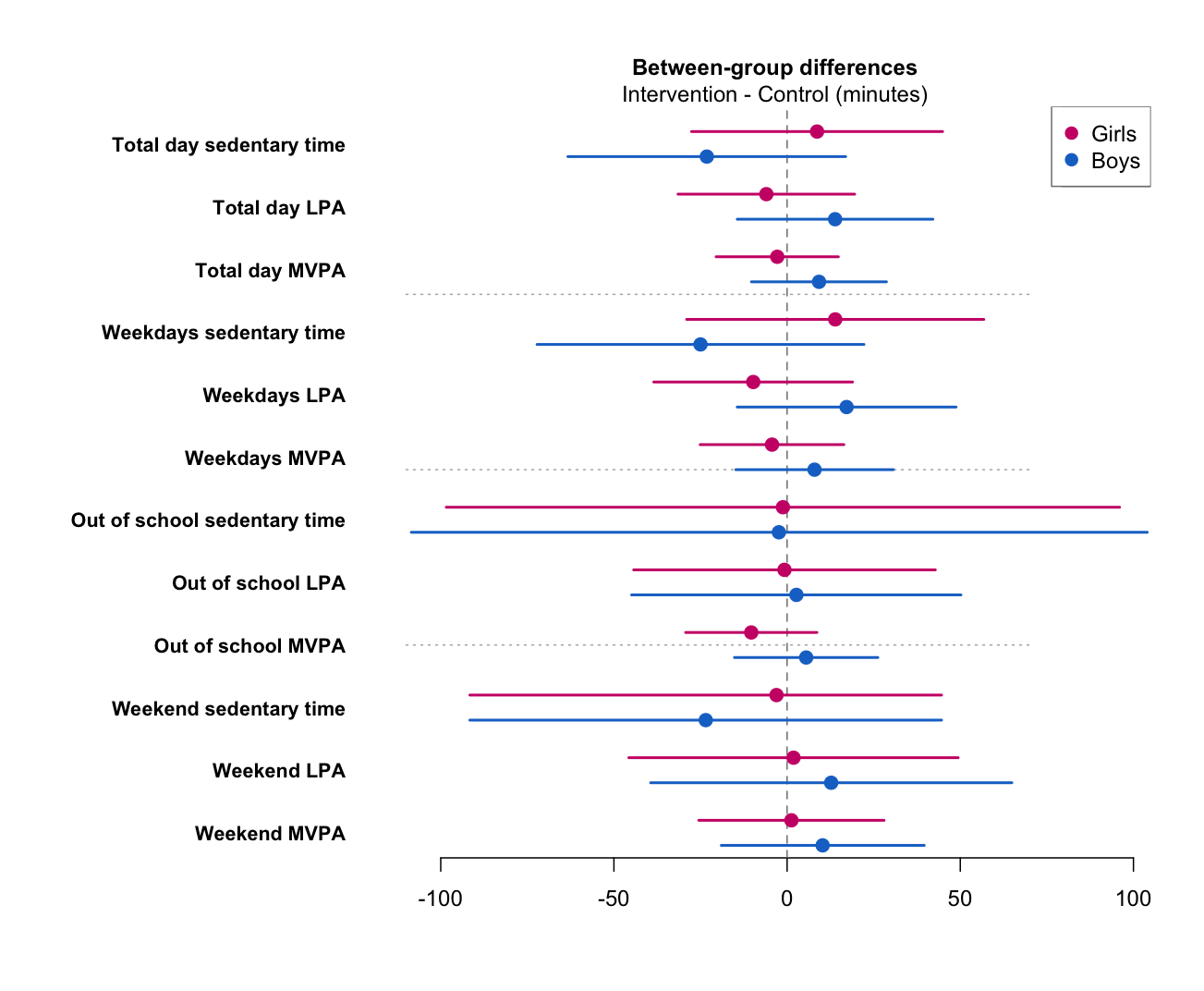


**Figure S12.** Per-protocol moderation effect of gender over the intervention effect on device-measured sedentary time, LPA, and MVPA during total day, weekdays, out of school, and weekend days. Values represent mean differences with 95% confidence intervals for girls (pink) and boys (blue). Positive values indicate higher minutes in the intervention group compared with the control group.

Adjusted for: bike in good condition, SES, and home-school distance.

**LPA** = light physical activity; **MVPA** = moderate-to-vigorous physical activity

**
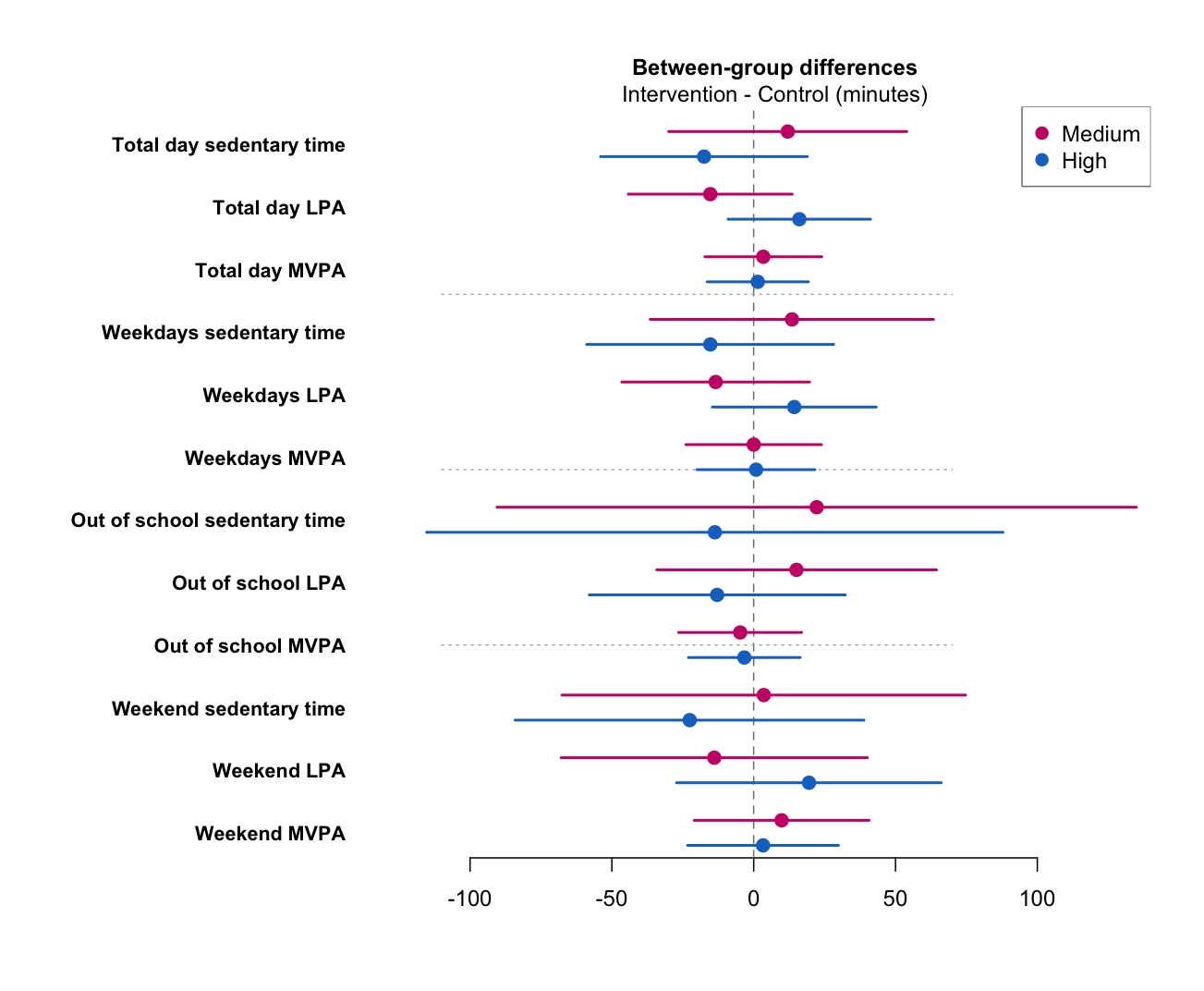
**

**Figure S13.** Per-protocol moderation effect of SES over the intervention effect on device-measured sedentary time, LPA, and MVPA during total day, weekdays, out of school, and weekend days. Values represent mean differences with 95% confidence intervals for medium (pink) and high (blue). Positive values indicate higher minutes in the intervention group compared with the control group.

Adjusted for: gender, bike in good condition, and home-school distance.

**LPA** = light physical activity; **MVPA** = moderate-to-vigorous physical activity; **SES** = socioeconomic status
